# Mutations in *Drosophila Greatwall/Scant* Reveal Its Roles in Mitosis and Meiosis and Interdependence with Polo Kinase

**DOI:** 10.1371/journal.pgen.0030200

**Published:** 2007-11-09

**Authors:** Vincent Archambault, Xinbei Zhao, Helen White-Cooper, Adelaide T. C Carpenter, David M Glover

**Affiliations:** CRUK Cell Cycle Genetics Group, Department of Genetics, University of Cambridge, Cambridge, United Kingdom; National Institute of Diabetes and Digestive and Kidney Diseases, United States of America

## Abstract

Polo is a conserved kinase that coordinates many events of mitosis and meiosis, but how it is regulated remains unclear. *Drosophila* females having only one wild-type allele of the *polo* kinase gene and the dominant *Scant* mutation produce embryos in which one of the centrosomes detaches from the nuclear envelope in late prophase. We show that *Scant* creates a hyperactive form of Greatwall (Gwl) with altered specificity in vitro, another protein kinase recently implicated in mitotic entry in *Drosophila* and *Xenopus*. Excess Gwl activity in embryos causes developmental failure that can be rescued by increasing maternal Polo dosage, indicating that coordination between the two mitotic kinases is crucial for mitotic progression. Revertant alleles of *Scant* that restore fertility to *polo–Scant* heterozygous females are recessive alleles or deficiencies of *gwl;* they show chromatin condensation defects and anaphase bridges in larval neuroblasts. One recessive mutant allele specifically disrupts a Gwl isoform strongly expressed during vitellogenesis. Females hemizygous for this allele are sterile, and their oocytes fail to arrest in metaphase I of meiosis; both homologues and sister chromatids separate on elongated meiotic spindles with little or no segregation. This allelic series of *gwl* mutants highlights the multiple roles of Gwl in both mitotic and meiotic progression. Our results indicate that Gwl activity antagonizes Polo and thus identify an important regulatory interaction of the cell cycle.

## Author Summary

Coordination of cell division in development requires a complex interplay between protein kinases, which catalyze the transfer of phosphates to specific substrate proteins to modify their activities. One of these kinases is the conserved Polo, which is the target of anticancer drugs. Using genetics in *Drosophila* (the fruit fly), we have identified Greatwall, another conserved protein kinase, as an antagonist of Polo. Studies of *Scant*, a dominant mutation of the *greatwall* gene, lead us to examine the effects of overexpressing wild-type Greatwall. Too much Greatwall activity relative to Polo leads to developmental defects in early syncytial embryos, which are initiated by the detachment of a single centrosome from the nuclear envelope in prophase. Loss-of-function mutants of *greatwall* reveal that the kinase is required for proper chromosome structure and segregation in mitosis and meiosis. One of these mutations results in the loss of Greatwall specifically during vitellogenesis (building up the egg's contents) and leads to a failure of meiosis I characterized by the premature loss of sister chromatid cohesion. This study shows that the Greatwall kinase fulfils multiple crucial functions in the different cell cycles of a developing animal and will be the foundation for further investigations.

## Introduction

Reversible protein phosphorylation and periodic protein destruction play major roles in regulating the eukaryotic cell division cycle. The major protein kinase that directs cell division is cyclin-dependent kinase 1 (Cdk1), the active component of Maturation Promoting Factor, first found to promote meiotic entry in amphibian oocytes. The cyclical inactivation of Cdk1 prior to mitotic exit is brought about in part through destruction of its cyclin partner (reviewed by [1]). Two other protein kinase families, the Polo and Aurora families, are known to have critical functions in progression into and through M phase (mitosis and cytokinesis) and functionally interact with each other and also with Cdk1 to mediate their functions.

Polo, originally discovered in *Drosophila* [2,3], exemplifies an evolutionarily conserved mitotic protein kinase. Polo, as well as its close orthologs, has been shown to function in multiple events essential for cell division. Polo was initially found to be essential for centrosome maturation and separation [2]. It promotes recruitment of the γ-tubulin ring complex and phosphorylates Asp to facilitate nucleation of an increased number of dynamic microtubules on mitotic entry (reviewed by [4]). At the G2/M transition, Polo (Polo-like kinase 1 in vertebrates) phosphorylates and activates the Cdc25 phosphatase responsible for removing inhibitory phosphates on Cdk1; this promotes mitotic entry [5]. It also functions at the kinetochore-microtubule interface to monitor tension; the 3F3/2 phospho-epitope seen on kinetochores in the absence of tension is a consequence of Plk1/Plx1 kinase activity in vertebrates [6,7]. Removal of cohesins from chromosomal arms in mitosis and meiosis also requires phosphorylation of cohesin subunits by Polo kinases (reviewed by [8]). In *Drosophila* meiosis II, Polo phosphorylates and inactivates the centromeric cohesion protector protein Mei-S332 [9]. In addition, Polo is required for cytokinesis [10]. The growing list of Polo kinase substrates is evidence of its role in multiple mitotic events.

It is clear that protein kinases such as Cdk1 and Polo are only part of a large network of protein kinases that regulate cell cycle progression, many of which are as yet poorly characterized. A genome-wide survey found that up to one-third of the protein kinome of *Drosophila* has some cell cycle role [11]. Depletion of the Gwl kinase from S2 cells by RNA interference (RNAi) led to a mitotic delay characterized by formation of long spindles and scattered chromosomes [11]. Yu and colleagues (2004) also found a mitotic role for Gwl kinase by characterizing missense hypomorphic mutants. Reduced *gwl* function results in mitotic defects in larval neuroblasts and tissue culture cells, including delay between late G2 and anaphase onset and chromosome condensation defects. Gwl has close homologs across eukaryotes and more distant homologs in budding and fission yeasts. Indeed, Yu and colleagues recently reported a function for *Xenopus* Gwl in mitotic entry, as part of the Cdc2/Cdk1 activation loop in oocyte extracts [12]. In that system, *Xenopus* Gwl is directly activated by cyclin B-Cdc2 and is in turn needed to promote full activation of cyclin B-Cdc2, although the direct target(s) mediating this action is (are) still unknown and indeed no substrates of Gwl are yet known. The primary sequence of Gwl shows that the regions most homologous to other kinases are split by a long intervening sequence of unknown function [13]. Despite this recent progress, nothing is known about how activity of this crucial kinase is coordinated with the multiple events of cell cycle progression. Moreover, it is not known how Gwl contributes to the different types of mitotic and meiotic cell cycles of a metazoan.

Elucidation of protein function may be aided through the generation of multiple mutant alleles that can reveal separate functions of individual proteins in multiple cellular processes. *Drosophila* offers the possibility of such studies and, moreover, allows the study of protein function in different types of cell cycle during its development. We applied this principle to study the gene defined by *Scant* (*Scott of the Antarctic*), a gain-of-function, dominant enhancer of maternal-effect embryonic defects of *polo* mutants. Syncytial embryos derived from females heterozygous for both *Scant* and *polo* develop mitotic defects in which a centrosome disassociates from one pole [14]. Here we report that the *Scant* mutation is an allele of *gwl* that introduces a K97M amino-acid substitution into the Gwl protein; this results in a hyperactive kinase with altered specificity in vitro. Our results indicate an antagonistic relationship between Gwl and Polo and suggest that their activity has to be coordinated for proper embryonic mitotic function. An allelic series of new *gwl* mutations reveals multiple functions for the Gwl kinase in both mitosis and female meiosis. These display somatic developmental defects accompanied by chromosome condensation and segregation defects in larval neuroblasts. *gwl^+^* encodes two isoforms, only one of which is expressed during vitellogenesis. An allele that specifically prevents the expression of this isoform reveals requirements for Gwl in meiosis and in the maternal contribution to the egg.

## Results

### *Scant*, a Dominant Enhancer of *polo* Maternal Effect Lethality, Is an Allele of *greatwall*

*Scant* is an ethyl methanesulfonate-induced mutation on the right arm of Chromosome III that causes greatly reduced female fertility when heterozygous with *polo^1^* (on 3L). These females produce embryos that develop characteristic mitotic abnormalities [14]. *Scant* shows no interaction with other mutants known to affect progression through embryonic cycles, namely *gnu*, *mgr*, *asp*, and *stg* [14]. The original *Scant*-bearing chromosome is also recessive female sterile [14]; however, although they are close, we were able to separate the dominant *Scant* from the recessive(s) by crossing-over (see Text S1). One recombinant that retained the dominant *Scant* interaction but was recessive female fertile was recovered; this and its derivatives were used in all the experiments reported here.

**Figure 1:**
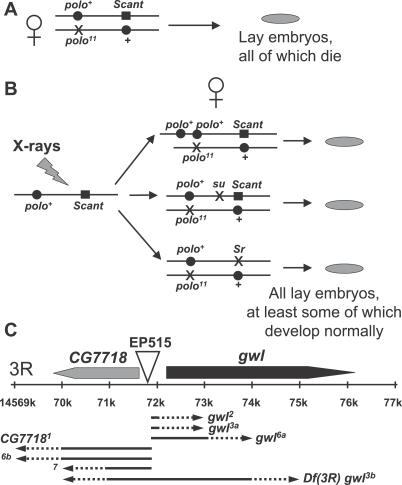
Generation of *gwl* Alleles (A) Females heterozygous for both the *Scant* mutation and a loss-of-function *polo* mutation (such as *polo^11^*) lay embryos that die during development. (B) Genetic screen to generate Scant revertant (*Sr*) alleles. Males homozygous for *Scant* were x-rayed and crossed to *polo^11^* heterozygous females. Female progeny were tested for fertility. Scant revertant mutations restore female fertility and can be duplications of the *polo^+^* gene, third-site suppressor mutations (*su*), or recessive mutations allelic to each other. See Text S1 for details. (C) Hopping a *P*-element inserted directly upstream of the *gwl* gene generated imprecise excisions disrupting either *gwl* (*gwl^2^, gwl^3a^*, and *gwl^6a^*), *CG7718* (*CG7718^1^, CG7718^6b^*, and *CG7718^7^)* or both genes (*gwl^3b^)*. *gwl^2^, gwl^3a^*, and *gwl^6a^* are semilethal and produce a “Scant-revertant (Sr) phenotype” when placed over *Sr* alleles (generated in [B]). The extent of the imprecise excisions was approximately mapped by the ability to PCR-amplify the 1 kb-long regions of genomic DNA defined by the tick marks.

*Scant* is a dominant enhancer of *polo* female sterility for all *polo* alleles, though the strength of the interaction varies with the defectiveness of the *polo* allele. *Scant* was originally described in combination with *polo^1^* [14], a hypomorphic allele; *polo^1^* homozygotes are viable though females are completely sterile. Typically, *polo^1^ +/+ Scant* females produce 4% as many progeny as controls, but *polo^11^ +/+ Scant* and *Df(3L)rdgC-co2, polo^−^ +/+ Scant* females are completely sterile (Figure 1A). *polo^11^* is a breakpoint allele, *In(3L)polo^11^*, 77E1–3;77E1–2 (see Text S1), and therefore probably amorphic; homozygotes are prepupal lethal. This suggests that the interaction depends on how much functional maternal Polo protein is available in the egg. However, the reciprocal question—whether the interaction depends on how much functional Scant protein is in the egg—cannot be asked; *Scant* is not only homozygous viable and fertile, it is viable and fertile over all deficiencies of its region, and none of these deficiencies affect fertility of heterozygous *polo* mutations. This suggests that the *Scant* mutation is hypermorphic or neomorphic. If it is, then the dominant interaction with *polo* mutations should be alleviated by mutations that inactivate its gene, and those mutations may have recessive phenotypes that can be deficiency mapped. We therefore recovered *Scant* revertants by mutagenizing homozygous males with X rays, mating them to *polo^11^/Balancer* females, and testing the + Scant*/polo^11^ + daughters for fertility. We recovered three classes of revertants (Figure 1B):

The first class is duplications of *polo^+^*: we recovered two, a tandem duplication and a 3;3 duplication transposition. This confirms the deduction from the genetic analysis that, in the presence of the hyper- or neomorphic *Scant* mutation, two maternal doses of *polo^+^* are needed for full female fertility.

The second class is a second-site suppressor because it is a 3L deficiency and independent overlapping deficiencies of its region also suppress the *polo*–*Scant* interaction; we have yet to identify the relevant gene.

The third class is represented by two recessive mutants in this first mutagenesis; they fail to complement each other genetically and were named Scant revertants (*Sr3* and *Sr6*; see Text S1). The chromosome carrying the second-site suppressor complements the recessive phenotypes of the third class.

*Sr3* is homozygous viable with eclosion delay; the adults have variably rough eyes, eroded wing margins, and missing bristles, all symptoms of mitotic defects, and both sexes are sterile with strongly reduced gonads. There are few germ cells even in newly eclosed adults; none remain a few days later. *Sr6* homozygotes die as pupae but this reflects a second lethal on its chromosome; *Sr3/Sr6* adults eclose, with the above constellation of phenotypes, though viability is reduced. Viability of *Sr3* and *Sr6* is further reduced over noncomplementing deficiencies, so the allelic series is *Sr3* (hypomorph), *Sr6* (more defective hypomorph), deficiency (see below). We sequentially uncovered the two recessive *Sr* alleles by three deficiencies: the first extends from 91A8 to 91F5; the second from 91B4 to 91D3; and finally between genome sites 14566000 and 14750000 (*Df(3R)Exel6181, *91C5;91D5). This restricts *Scant* to one of 16 potential genes in an interval of 185 kb.

To further refine the *Sr* mapping, we mobilized four different viable *P*-element insertions in or near this 185-kb region to generate imprecise excisions (Figure 1C; Text S1) with delta 2–3 transposase. All progeny receiving the hopping *P*-element chromosome were crossed either to *Df(3R)Exel6181* or *Sr3*. This allowed us to determine whether a lethal excision had occurred or whether an excision or hop failed to complement the Scant revertant. One particular line, *gwl ^EP515^*, whose *P* element is inserted 217 bp 5′ of *gwl* and 12 bp 5′ of *CG7718*, yielded several hits (Figure 1C). Complementation tests define three groups of mutations: (1) three independent events *(gwl^2^*, *gwl^3a^*, and *gwl^6a^)* that fail to complement *Sr3*; (2) three independent events *(CG7718^1^*, *CG7718^6b^*, and *CG7718^7^)* that complement *Sr3* but fail to complement each other; and (3), one event *(gwl^3b^)* that fails to complement mutations of both groups. PCR revealed that the *gwl* gene is disrupted in mutations of the first group from imprecise excision extending in one direction. *CG7718* is disrupted in the second group from imprecise excision extending in the other direction. Finally, both *gwl* and *CG7718* are disrupted in the third event; the imprecise excision extends in both directions. The failure of *gwl^2^*, *gwl^3a^*, and *gwl^6a^* to complement Scant revertant alleles suggests that the *Scant* mutation affects the *gwl* gene; this was confirmed by sequencing *gwl* in them (see “*Scant* Encodes a Hyperactive Gwl Kinase with Altered Specificity” and “The Allelic Series of Recessive *gwl* Alleles Reveals Multiple Mitotic Functions” below). *gwl^2^* and *gwl^3a^* are probably deletions into *gwl*, and *gwl^6a^* clearly is; both 5′ upstream sequence and N-terminal coding sequence have been deleted. All three of these small deletions give escapers homozygous and over deficiencies on nutritious food, with the *Sr* constellation of phenotypes. Thus, the *gwl* gene is not absolutely vital.

Finally, we carried out a second X-ray mutagenesis, now of a *polo^11^ Scant* recombinant chromosome made from the fertile *polo^11^/Dp(polo^+^) Scant* females and tested over a third-chromosome balancer (Figure S1). This gave ten positives, all of class three; four are cytologically visible deficiencies each of which removes all or part of 91C5–6 (plus more), one is a translocation with one breakpoint in 91C, and the remaining five have no obvious relevant cytological defect. For two of these, all or part of the *gwl* gene fail to PCR up, so they are probably additional deletions. All seven of these fail to complement *Sr3* for both phenotype and viability. The final three, *Sr17*, *Sr19*, and *Sr18*, complement *Sr3* and deficiencies for phenotype but females have reduced fertility or are sterile. *Sr17* and *Sr19* also have reduced viability over *Sr3* in the presence of *polo^11^* and some DNA defect 5′ of the *gwl* coding region (probably an insertion based on PCR, see Text S1). Both alleles reduce the amount of Gwl protein produced but have not been studied further. *Sr18* is perfectly viable but is absolutely maternal-effect female sterile; this allows us to separate somatic *gwl* function from its role in producing a functional egg. All further studies of *Sr18* used a chromosome from which *polo^11^* had been removed by recombination.

Thus we identified three categories of *gwl* alleles: the dominant *Scant* allele, recessive zygotic plus germline alleles, and a recessive maternal-effect allele. Our characterization of these three groups gives new insights into the mitotic and meiotic functions of the protein kinase that *gwl* encodes.

### *Scant* Enhances *polo* Defects at Spindle Poles

The severity of the dominant *Scant* mutant phenotype increases in relation to the decrease in *polo* function. Not only is the dominant effect overcome by two doses of *polo^+^* in the presence of a *polo* mutation (see above), but also the severity of the phenotype in the presence of one copy of *polo^+^* is proportional to the strength of the *polo* allele. Weak hypomorphic alleles such as *polo^1^*, a viable allele that shows maternal effect lethality, give some progeny when heterozygous with *Scant*. Amorphic alleles such as *polo^11^* (see above) and *polo* ^−^ deficiencies are completely sterile; these females lay normal numbers of eggs that do not hatch but do begin to develop and turn brown.

**Figure 2:**
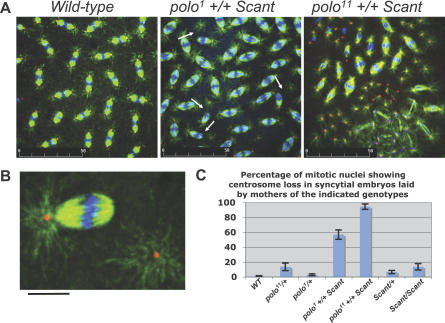
*Scant* Interacts Genetically with *polo*, Leading to Mitotic Defects in Embryos (A) Aberrant mitotic figures in embryos derived from females heterozygous for *polo* and *Scant*. Embryos were collected for 2–3 h, dechorionated, and fixed for immunofluorescence. Stainings are α-tubulin (green), γ-tubulin (red), and DNA (blue). Representative examples of mitotic phenotypes are shown for the indicated genotypes. Arrows in the center panel indicate the displacement of centrosomes from one pole. Scale bars are in μm. (B) Typical displacement of one centrosome observed in embryos derived from *polo^1^ +/+ Scant* heterozygous females. The scale bar represents 10 μm. (C) Quantitation of aberrant mitotic figures observed. Embryos were treated as in (A) and syncytial single embryos with mitotic nuclei were scored for the percentage of defective nuclei (having lost at least one centrosome). Numbers are average percentages (± standard error of the mean).

The archetypal *Scant* phenotype is shown by embryos derived from *polo^mut^ +/*+ *Scant* females. The mitotic figures of such embryos frequently display centrosome disassociation from one pole (Figure 2A and 2B). To compare severity of phenotypes we counted the number of defective mitotic nuclei (showing detachment of at least one centrosome) in syncytial embryos derived from mothers of different genotypes (Figure 2B and 2C). There is a slight but significant increase in defective spindles in embryos derived from *polo^1^/+*, *polo^11^/+*, *Scant/+*, and *Scant/Scant* females relative to wild-type females, indicating that a single mutant copy of these genes in mothers leads to mitotic defects at a low frequency (Figure S2). However, these embryos always hatched and developed fully, indicating that such low frequencies of defects can be tolerated. The mitotic spindles in embryos derived from *polo^11^ +/+ Scant* females are more frequently aberrant than those derived from *polo^1^* +/+ *Scant* females. Thus the severity of the maternal effect phenotypes observed at the cellular level is consistent with the relative strengths of the *polo* alleles as homozygotes, indicating that, in the context of the early embryo, *Scant* enhances mitotic defects resulting from a decrease in *polo* function.

**Figure 3:**
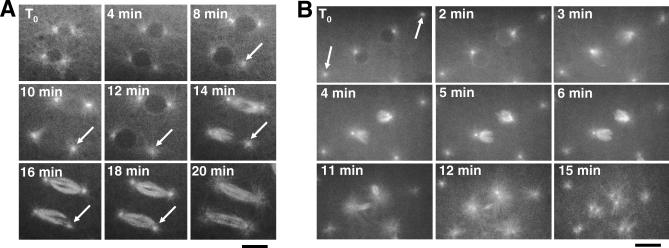
Centrosome Dissociation Is Observed in Prophase in *polo-Scant*-Derived Embryos Time-lapse fluorescence microscopy of embryos derived from *GFP-β-tubulin; polo^11^ Scant/+ +* mothers. In both (A) and (B), centrosomes dissociate prior to nuclear envelope breakdown. (A) In some cases, the disassociated centrosome (arrow) can be recaptured and mitosis can proceed normally. (B) In other cases, centrosomes are lost irreversibly and gross mitotic defects develop. Both series (A and B) were taken from Video S2. Scale bars correspond to 10 μm.

To examine the formation of the aberrant mitotic figures in *polo^11^ Scant*-derived embryos, we used time-lapse microscopy to follow mitosis in embryos that also expressed green fluorescent protein (GFP)-β-tubulin constitutively from the ubiquitin promotor (Figure 3; Videos S1 and S2). No defects were observed in embryos derived from the wild-type GFP-β-tubulin stock (Video S1). In contrast, *polo^11^ Scant/+ +*-derived embryos show an initial detachment of one centrosome early in mitosis, before nuclear envelope breakdown. All 33 cases where the filming was continuous from before the centrosome detached show that the detachment occurs prior to nuclear envelope breakdown and involves only one centrosome. The free centrosome drifts away from the nucleus, and astral microtubule formation usually appears normal, though there is no asymmetric microtubule enhancement. A half-spindle is established by microtubules forming connections between the chromosomes and the centrosome still associated with the nuclear envelope. However, spindle bipolarity is often attained by microtubules growing from the chromosomes outwards. If a free centrosome is sufficiently close to this second half-spindle, it can reattach to it to form a normal bipolar spindle containing two centrosomes and nuclear division completes normally (Figure 3A; Video S2). However, if the free centrosome drifts too far away from its spindle, it cannot be recaptured, and the monoastral spindle that forms initially is unfocussed at the pole lacking a centrosome. In some cases, monoastral bipolar spindles fuse with neighboring spindles (Figure 3B; Video S2) and degenerate to give interconnecting arrays of microtubules (Figure 2A). In other cases, the acentrosomal pole eventually focuses and anaphase occurs (Video S2) (unpublished data) as previously observed in *sak* mutants, which lack centrosomes [15]. The primary defect in these mitoses is therefore premature centrosome detachment; subsequent spindle abnormalities reflect secondary, mechanical problems. Somehow the Polo-Gwl kinase balance is important for maintaining centrosome-nuclear envelope propinquity until nuclear envelope breakdown.

Free centrosomes always show the presence of γ-tubulin, pericentrin-like protein (PLP) and centrosomin (CNN) when the attached one does, while the acentrosomal spindle poles always lack all three antigens (unpublished data)—free centrosomes seem to mature normally. Indeed, free centrosomes are mature by the functional test as well, since they can serve as active poles if they become reassociated with a spindle (Figure 3A).

Loss of centrosomes in *Drosophila* embryos has been shown to occur in response to DNA damage; nuclei then drop from the cortex into the interior of the syncytial embryo [16]. Both centrosome loss and nuclear fallout are suppressed in embryos lacking the Chk2 protein kinase [17]. Centrosomes still detach in embryos derived from *chk2/chk2; polo^11^ Scant/+ +* females (Figure S3), suggesting that the loss of centrosomes in embryos laid by *polo^11^ Scant* females is not the consequence of DNA damage inducing its response pathway. This independence from Chk2 as well as the enhancement of *polo* phenotypes by the *Scant* mutation suggests that coordinated activity of the protein kinases encoded by these genes is required directly to coordinate centrosome attachment to the nuclear envelope before spindle assembly.

### *Scant* Encodes a Hyperactive Gwl Kinase with Altered Specificity

To identify the nature of the *Scant* mutation, we sequenced *gwl* on both the *Scant* chromosome and on several non-*Scant* mutant chromosomes that resulted from the same mutagenesis [14]. The sole difference is an A to T base change in *Scant*, which changes amino acid residue 97 from lysine to methionine. The *gwl* sequence in the *Sr* mutations retains the K97M substitution codon as well as additional changes, consistent with their recessive reduction in function (see “The Allelic Series of Recessive *gwl* Alleles Reveals Multiple Mitotic Functions” below).

**Figure 4:**
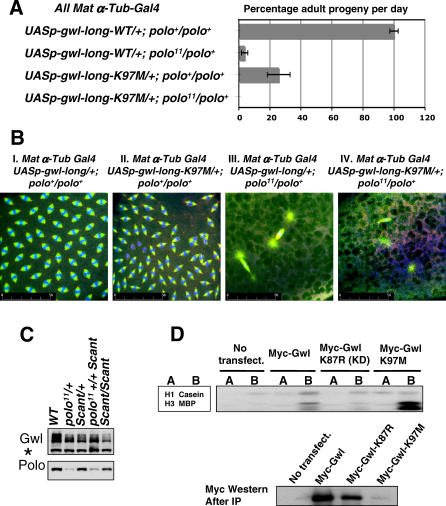
Gwl-K97M Is Hyperactive (A) Effects of overexpressing *gwl-long-wt* or *gwl-long*-*K97M* (*Scant*) on female fertility. *Mat* α*-Tub-Gal4* and the *UASp-gwl* transgenes were present in only one copy on the same chromosome in all flies tested. Single females of the indicated genotypes with 3 *WT* (*Oregon R)* males laid eggs for 3 consecutive d. Numbers are averages of hatched adult progeny (± standard error of the mean) per day for 12 females of each genotype (three females of each of four independent transgenic lines per genotype = 36 samples). The 100% reference comes from the observation of 100% egg hatch and no larval or pupal lethality. (B) Effect of overexpressing *gwl-long-wt* or -*K97M* (*Scant*) on embryonic mitosis. Embryos were laid by mothers of the indicated genotypes and treated as in Figure 2A. Scale bars are in μm. (C) Endogenous Scant protein is not more abundant than Gwl-wt in embryos. Western blots for Gwl and Polo from embryos from mothers of the indicated genotypes. *, cross-reactive band that serves as a loading control. (D) Gwl-K97M is hyperactive in vitro with altered specificity. Myc-tagged forms of Gwl-wt, K87R (kinase dead), and K97M (Scant) were expressed in Dmel stable cell lines. Myc immunoprecipitations were carried out and the kinase activity on Histones H1, H3, Casein, and Myelin Basic Protein was assayed on beads. Note that the Gwl-K97M was always expressed at lower levels in stable cell lines, suggesting toxicity for this protein.

To confirm that the K97M substitution in Gwl is indeed responsible for the *Scant* phenotype, we reconstituted the genetic interaction with *polo* using a synthetic *gwl-K97M* generated in a wild-type *gwl* sequence and carried as a transgene. Because the *Scant* mutation appears to be hyper- or neomorphic, we also asked whether expressing wild-type (wt) Gwl kinase at higher levels than normal generates the characteristic embryonic mitotic defects from *polo/+* mothers*.* There are two isoforms of Gwl; the female vitellogenic ovary expresses only the long form (see “The Long Isoform of *Gwl* Is Required for Female Meiosis and Is Provided to the Egg” below). We therefore made transgenic flies expressing either the longer isoform of Gwl-Wt or Gwl-K97M (otherwise identical to wild type) under the control of the *UASp* promotor, driven by Gal4 expressed from the *maternal* α*-tubulin* promotor (*Mat* α*-Tub Gal4*). Expression of the transgenes (checked by western blot; unpublished data) was driven at comparable levels in the germline in *polo^11^*/*polo^+^* and *polo^+^*/*polo^+^* females, and in both cases the transgenic expression exceeds the endogenous Gwl by approximately 3-fold (unpublished data). Female germline expression of Gwl-K97M in *polo^11^/polo^+^* heterozygotes causes complete sterility (Figure 4A). These embryos show loss of centrosomes from very early mitotic spindles (Figure 4BIV), a much stronger and earlier phenotype than *Scant* itself. Overexpression of Gwl-wt in heterozygous *polo^11^/polo^+^* females allows partial egg hatch (around 4%), and these embryos also show loss of centrosomes from spindles in early syncytial divisions (Figure 4BIII). Fertility is also reduced (to 23%) in *polo^+^/polo^+^* females expressing Gwl-K97M where centrosome loss usually occurs in the later divisions of the syncytial blastoderm stage (Figure 4BII). However, *polo^+^/polo^+^* females overexpressing Gwl-wt are fully fertile and their embryos do not show significant centrosome detachment (Figure 4BI). Thus embryonic mitotic defects and maternal-effect lethality arise from overexpression of either the wild-type or *K97M* mutant forms of Gwl kinase in the female germline when Polo kinase function is reduced, but only the K97M form induces a phenotype when Polo function is normal.

The above results show that the *Scant* phenotype in embryos with compromised Polo function can arise just from increased maternal expression of Gwl kinase but also indicate that the Gwl-K97M protein has additional properties, perhaps increased stability or altered activity. Western blots of *gwl^+^* and *Scant*-derived embryos show no increase in levels of Gwl resulting from the *Scant* mutation; if anything there is less Gwl in homozygous *Scant*, so an increase in stability is unlikely (Figure 4C). To ask whether *Scant*'s K97M mutation affects the kinase activity of Gwl, we expressed Myc-tagged forms of Gwl (WT or K97M or kinase-dead [K87R]) in *Drosophila* cells in culture and purified the fusion proteins for in vitro kinase assays (Figure 4D). Myc-Gwl phosphorylates myelin basic protein and casein more efficiently than histones H1 or H3, although the signals are only marginally increased above the K87R, presumably kinase-dead, control (the K87R mutation may not effectively abolish the kinase activity or phosphorylation could result from traces of copurifying kinases). Myc-Gwl-K97M specifically phosphorylates myelin basic protein with dramatically increased efficiency, while showing only slight increases for casein and H1 and no increase for H3. This high activity on myelin basic protein was repeatedly observed despite the lower amount of kinase present in the reaction for Myc-Gwl-K97M compared with Myc-Gwl or Myc-Gwl-KD. Indeed we found that Gwl-K97M was reproducibly expressed at a lower level than its WT equivalent in several independent stable cell lines. This suggests that the hyperactive K97M mutation is toxic to the cell line used, so the only stable transformants that survive are those that can keep it downregulated. Both our genetic and biochemical results show that the *Scant* mutation (Gwl-K97M) results in a hyperactive enzyme with altered specificity.

Taken together, several pieces of evidence indicate that Gwl and Polo have antagonistic activities in the early embryo. The *Scant* phenotype is not seen in the presence of wild-type levels of *polo*, and its strength is dependent upon the strength of the mutant *polo* allele. The failure of embryos to develop upon overexpression of overactive Gwl-K97M and, to a lesser extent, of Gwl-wt, is strongly dependent on reduced Polo dosage. Two of the mutations that restore fertility to *polo-Scant* heterozygous females are duplications of *polo^+^* (Figure 1B; Text S1). These results suggest negative regulatory interactions between Gwl and Polo and indicate that the balance between these two protein kinase activities is paramount for success of the rapid mitotic cycles of early embryonic development.

### The Allelic Series of Recessive *gwl* Alleles Reveals Multiple Mitotic Functions

**Figure 5:**
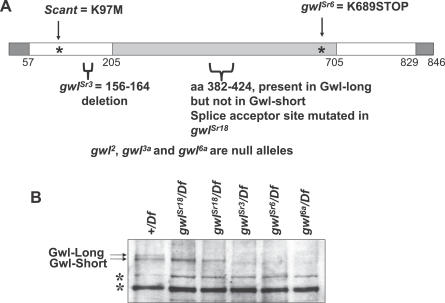
*gwl* Alleles Generated and Used in This Study (A) Molecular map of *gwl* alleles. Gwl's highly conserved kinase domain (white) is predicted to be split by a less-conserved intervening sequence of yet unknown function (light gray). The N-terminal and C-terminal ends are also less conserved (dark gray). This diagram is inspired from the one proposed in Yu et al. [13]. The *Scant* mutation changes lysine residue 97 to methionine, making Gwl hyperactive and altering its specificity in vitro. Mutation *Scant revertant 3* (*gwl^Sr3^*) removes exactly nine codons coding for residues 156–164 predicted to be part of the kinase fold. Mutation *Scant revertant 6* (*gwl^Sr6^)* introduces a premature ochre termination codon instead of lysine 689. Genetically, both mutations *gwl^Sr3^* and *gwl^Sr6^* seem to have residual Gwl function. Mutation *gwl^Sr18^* changes the reading frame following the splice acceptor site at the beginning of exon 4, which encodes residues 382–424 in Gwl-long. Exon 4 is spliced out from Gwl-short and, therefore, Gwl-short is unaffected by the *gwl^Sr18^* mutation. Mutations *gwl^2^, gwl^3a^*, and *gwl^6a^* are null alleles generated by imprecise excisions of a *P*-element (see Figure 1C). (B) Anti-Gwl western blot on larval brain extracts of the indicated genotypes. Gwl-long and Gwl-short are both visible in the *WT* lane (+/*Df*). Two *gwl^Sr18^/Df* samples were loaded to confirm that the absence of the top Gwl band is not artifactual.

The hypomorphic recessive alleles of *gwl* allowed us to study the roles of the Gwl protein kinase in several of the different cell cycle types in *Drosophila* development. We compared their mutant phenotypes to those of the null allele *gwl^6a^*, a *P*-hop deletion that removes the N-terminal end of the Gwl protein and its 5′ upstream sequence (described above). Two close bands are detectable around 100 kDa in Gwl western blots from larval brains, and both bands are absent in hemizygous *gwl^6a^* larval brains (Figure 5B).

DNA sequencing revealed that all of the nondeficiency Scant revertant alleles (*Sr*) retained the K97M *Scant* mutation (Figure 5A). *gwl^Sr3^* has, in addition, a 27-bp deletion corresponding to amino acids 156–164 (Figure 5A). This short deletion maps to the predicted kinase fold in the N-terminal portion (not shown). The *gwl^Sr3^* allele is a hypomorph since *gwl^Sr3^/gwl^Sr3^* is more viable than *gwl^Sr3^/Df;* and *gwl^Sr3^/Df* is in turn more viable than *gwl^null^/Df*. This predicts that the kinase encoded by the *gwl^Sr3^* allele retains some activity. However, western blots of extracts of *gwl^Sr3^/Df* larval brains reveal little or no protein, suggesting that the protein encoded by *gwl^Sr3^* is unstable (Figure 5B). *gwl^Sr6^* has an A to T substitution that changes lysine 689 into the ochre termination codon (STOP; Figure 5A). *gwl^Sr6^* also behaves as a hypomorphic allele suggesting either that this STOP codon can be suppressed to some extent or that the truncated protein retains partial function. We see no Gwl protein in western blots of *gwl^Sr6^/Df* larval brain extracts (Figure 5B) but our anti-Gwl antibodies were raised against the C-terminal part of the protein that is predicted to be truncated in this mutant, so this test is unreliable. The weak hypomorphs *gwl^Sr17^* and *gwl^Sr19^* show a slight reduction in levels of both forms of Gwl in brains (unpublished data). Finally, the female-sterile-only allele*, gwl^Sr18^*, has one base deleted in the splice acceptor sequence of exon 4. This change in sequence from AAAGGGCT to AAAGGCT (Figure 5A) leaves the same splice-acceptor sequence but changes the reading frame after it to encode a string of 61 different amino acids followed by a series of STOP codons. This mutation has lost the slower migrating isoform of Gwl (Gwl-long) in neuroblasts of *gwl^Sr18^/Df* larvae without affecting the faster form (Gwl-short) (Figure 5B). Both isoforms of Gwl are also expressed in S2 cells (Figure S4) [13]. We cloned both Gwl cDNAs from total S2 cell mRNA; sequencing revealed that the shorter cDNA lacks exon 4 precisely. RNAi treatment of S2 cells using double-stranded (ds)RNA targeting exon 4 depleted the upper but not the lower Gwl band seen on western blots (Figure S4), whereas RNAi to common exons depleted both isoforms. Thus *gwl^Sr18^* has a mutation in a splice site used in the synthesis of only one of the two isoforms.

The mutations fall into an allelic series. The amorphic genotype *gwl^6a^/Df* dies predominantly in the pupal stage, and the rare escaper adults have rough eyes, ragged wings, cuticle defects, missing bristles, and are sterile, phenotypes typical of cell division cycle mutants. We found similar, but less severe, phenotypes in *gwl^Sr3^/Df*, *gwl^Sr6^/Df*, and *gwl^Sr3^/gwl^Sr6^* animals, a greater proportion of which survive to adulthood (Figure S5). In contrast, *gwl^Sr18^/Df* flies are fully viable and show no such morphological defects but females are sterile (males are fertile).

**Figure 6:**
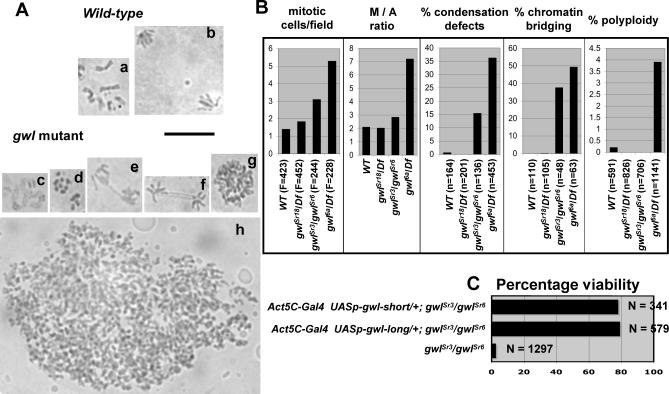
Gwl Is Required for Normal Mitosis in Somatic Tissues (A) Chromosomal defects and polyploidy observed in *gwl* mutant larval neuroblasts following orcein DNA staining. Control prometaphase (a) and anaphase (b) figures are shown, with normal condensation. Examples of undercondensation (c), overcondensation (d), anaphase bridges (e and f) and polyploidy (g and h) observed in *gwl* mutants are shown. The scale bar is for all images and shows 10 μm. (B) Quantitation of defects observed in larval neuroblasts for *WT* (control), *gwl^Sr18^* (female germline specific), *gwl^Sr3^/gwl^Sr6^* (partial loss of function), and *gwl^6a^/Df* (complete loss of function). Mitotic cells were recognized by their condensed chromatin. The M/A ratio corresponds to the number of prometaphase and metaphase cells over the number of anaphase and telophase cells (*F* = number of fields scored). % condensation defects, number of mitotic cells with under- or overcondensation/total number of mitotic cells (*n*); % chromatin bridging, number of bridged anaphases/total number of anaphases (*n*); % polyploidy, number of polyploid mitotic cells/total number of mitotic cells (*n*). (C) Rescue of *gwl^Sr3^/gwl^Sr6^* mutants by the expression of *UASp-gwl-long* or *UASp-gwl-short* from the ubiquitous *Actin5C-Gal4* driver. The percentages of expected adult progeny of the indicated genotypes if the viability was normal is shown (numbers at the end of each bar indicated the total number of internal reference progeny flies scored). See Text S1 for details.

The severity of developmental defects is paralleled by the cellular phenotype of third instar larval neuroblasts in the *gwl* mutants (Figures 6 and S6). Strikingly, the viable but female-sterile splice acceptor site mutant that expresses no long isoform, *gwl^Sr18^*, shows no significant mitotic defects. The other Scant revertant alleles (illustrated here by *gwl^Sr3^/gwl^Sr6^*) all show mitotic defects that are exaggerated in hemizygotes, and *gwl^Sr6^* is usually more affected than *gwl^Sr3^* (Figure S6). In Figure 6, the strongest phenotypes are seen in *gwl^6a^/Df*. There is an increase in the mitotic index; more cells are pre-anaphase (Figure 6B). RNAi targeted to the first exon of *gwl* in cultured S2 cells depletes both isoforms and also results in an increase in mitotic cells, confirming earlier findings [11]. These cells show BubR1 staining on kinetochores and high cyclin B levels (Figure S4), indicating that they are delayed in prometaphase. *gwl* mutant neuroblasts also show a high frequency of defective chromatin condensation. However, in contrast to the undercondensation reported in other *gwl* alleles by Yu and colleagues [13], we consistently observe that some regions of chromosomes appear undercondensed and other regions are overcondensed (Figure 6A). In some cells the chromosomes are uniformly overcondensed, suggesting lengthy pre-anaphase delay (Figure 6A). In S2 cells depleted of Gwl by RNAi, we do not observe strong undercondensation, but rather chromosomes are scattered along extensively elongated spindles (Figure S4), as also found by Bettencourt-Dias et al. [11]. The small number of cells that manage to enter anaphase in mutant neuroblasts frequently show anaphase bridges (Figure 6A). The *gwl* null *gwl^6a^/Df*, unlike the hypomorphs, has a significant proportion of polyploid mitotic figures (Figure 6A and 6B). Conditions that block anaphase, such as colchicine treatment, also induce polyploidy; eventually the hypercondensed metaphase chromosomes decondense, and the cell reenters interphase, bypassing the mitotic checkpoints. Since *gwl^6a^/Df* cells show significant pre-anaphase delay with hypercondensed chromosomes, polyploidy is in fact expected from this normal interphase reentry. The absence of polyploid cells in the hypomorph *gwl^Sr3^/gwl^Sr6^* suggests that enough active Gwl is available to complete mitosis normally—eventually.

Expression of *UASp-gwl-long* or *UASp-gwl-short* as transgenes driven ubiquitously by *Actin 5C-Gal4* rescues the viability and somatic integrity of *gwl^Sr3^/gwl^Sr6^*, *gwl^Sr3^/gwl^Sr3^*, *gwl^Sr3^/Df*, and *gwl^Sr6^/Df* flies (Figures 6C and S5) (unpublished data) to similar degrees. Therefore, both forms of Gwl (long and short) are active kinases that have redundant functions in somatic tissues where they are both present (Figure 5B).

### The Long Isoform of Gwl Is Required for Female Meiosis and Is Provided to the Egg

**Figure 7:**
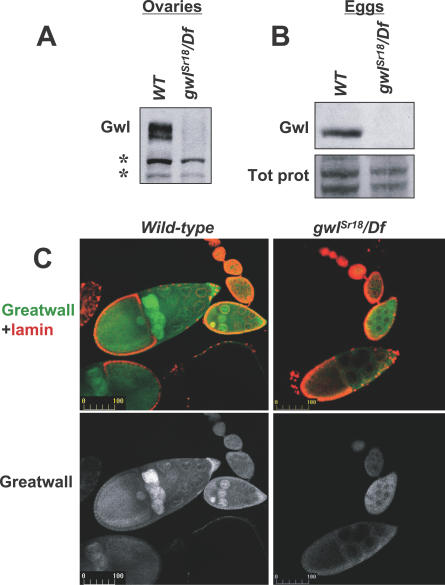
Gwl Is Supplied by the Mother to the Developing Egg (A) Anti-Gwl western blot from ovaries of *gwl^Sr18^/Df* or *WT (Oregon R)* females. (B) Anti-Gwl western blot from extracts of eggs laid by *gwl^Sr18^/Df* or *WT (Oregon R)* virgin females. Total proteins were stained by amido black, shown as a loading control. *, cross-reactive band acting as a loading control. (C) Localization of Gwl in ovaries. In addition to Gwl (green), lamin (red) was also stained to show the outlines of the cells and of their nuclei. In *WT* ovaries, Gwl is clearly visible in the nucleus and the cytoplasm of the oocyte and in the nuclei and cytoplasm of the four nurse cells connected to it. In *gwl^Sr18^/Df* ovaries, only follicle-cell staining of Gwl-short and/or background staining is detected. Scale bars are in μm.

*gwl^Sr3^*, *gwl^Sr6^*, and *gwl^6a^*, in which levels of Gwl are reduced (at least in neuroblasts; Figure 5B), are all sterile in both males and females. This sterility reflects cell-proliferation failure of the germlines and probably of their supporting somatic tissues as well. In contrast, *gwl^Sr18^/Df* shows normal viability and has no somatic defects including normal ovaries and testes, despite expressing only the short isoform of Gwl (Figure 5B). Therefore, the short form is sufficient for mitosis in general, and it is both necessary and sufficient for the mitotic divisions of germline cells in ovaries and testes. *gwl^Sr18^/Df* females lay lots of eggs but they remain white; males are fertile. Since *gwl^Sr18^* produces eggs, the Gwl-long isoform has a specific role late, rather than early, in germline function. Western blotting of mature wild-type ovaries or unfertilized eggs does show high levels of Gwl (Figure 7A and 7B); a single band in unfertilized eggs (Figure 7B) and a thick band in ovaries (Figure 7A). In contrast, *gwl^Sr18^/Df* females show a great reduction in Gwl signal in these tissues. Since our molecular analysis (cloning and sequencing) and biochemical analysis (in neuroblasts) reveal that only the long form is affected by the *gwl^Sr18^* mutation and since Gwl signal disappears from *gwl^Sr18^/Df* eggs and ovaries, the western signal in wild-type eggs and ovaries arises from the long form only. Gwl-short was not detected in eggs or mature ovaries, although Gwl-short must be present in *gwl^Sr18^/Df* ovarian follicle cells and premeiotic germline mitotic cells, since these do divide normally. Gwl-long appears as a thick band in western blots of wild-type ovary extracts (Figure 7A); this presumably corresponds to activated phosphoforms of Gwl-long analogous to those seen in [12], while it appears as a thin band in blots of unfertilized eggs (Figure 7B), where Gwl-long may not be activated.

When we examined the ovaries of *gwl^Sr18^/Df* females we found them fully developed (Figure 7C). However, preliminary observations reveal that late (stages 13–14) eggs often have irregularly distributed yolk, in no fixed pattern. Earlier stages have uniform yolk distribution but appear to have less of it than same-stage controls (our unpublished observations). The *Drosophila* ovary comprises 15 or so ovarioles each containing multiple egg chambers at successive stages of development. Cystoblasts in the germaria of the ovarioles divide four times to form cysts of 16 cells that remain interconnected by ring canals derived from the cleavage furrows of incomplete cytokinesis. Of the two cells interconnected by four ring canals, one will become the oocyte although formation of synaptonemal complex is complete in both of them. Once the oocyte is determined, the other 15 cells undergo endoreduplication cycles and associated cell growth to become the nurse cells. The morphology of egg chambers of *gwl^Sr18^*/*Df* females appears normal; they have 15 nurse cells and one oocyte indicating that the preceding mitoses had progressed normally. We detect strong staining for Gwl (the antibody recognizes both isoforms) in wild-type egg chambers from stage 8 of oogenesis onwards but not in *gwl^Sr18^*/*Df* females (the faint signal in *gwl^Sr18^*/*Df* ovaries is probably Gwl-short present in the follicle cells surrounding the egg chamber but some may be background). In wild-type egg chambers, Gwl protein is present in the oocyte and four neighboring nurse cells; actin staining shows that all four nurse cells connect directly to the oocyte by ring canals and that they have the expected numbers of ring canals to be the oocyte's sister and daughters (four, three, two, and one; unpublished data). In each of these cells Gwl accumulates in the nucleus, although there is some cytoplasmic staining. The long isoform of Gwl is contributed maternally to the embryo where it also concentrates in interphase syncytial nuclei. During mitosis Gwl is depleted in the nucleus and enriched around the outside of the spindle envelope before accumulating in the nucleus once again during the next interphase (Figure S7). Thus Gwl-long accumulates in a subset of polyploid nuclei of nurse cells, the prophase I nucleus of the oocyte and interphase nuclei of the embryo.

**Figure 8:**
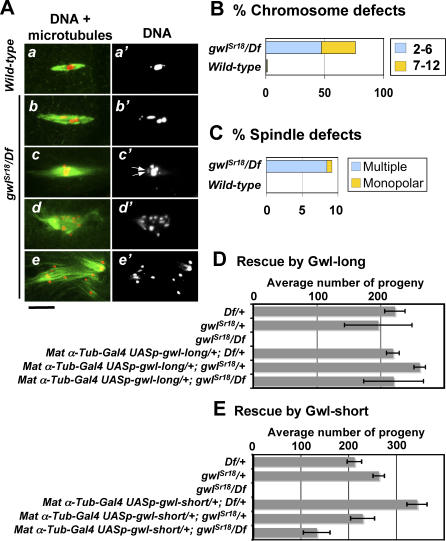
Gwl Is Required for Female Meiosis (A) Examples of meiotic defects observed in *gwl^Sr18^/Df* oocytes. Alpha-tubulin is stained green and DNA is red. Note the small, probably nondisjoined Chromosomes 4 in b-b'. In c', arrows indicate likely separated sister chromatids of the fourth chromosome. A typical wild-type (*Oregon R*) meiotic figure arrested in metaphase I is shown for comparison (a). The longer spindles in b and c are a typical feature of the bipolar spindles formed in *gwl^Sr18^/Df* oocytes. Scale bar is 10 μm. (B) Quantitation of chromosomal defects observed in metaphase and anaphase oocytes. In wild-type oocytes, only 2% appear to have progressed into a normal anaphase (not included in this quantitation). Chromosome defects were characterized by scattered chromosome masses. The number of DNA masses, excluding the tiny fourth chromosome, was counted in defective figures. The percentage of defective figures presenting two to six DNA masses (as in Ab–c) or seven to 12 DNA masses (as in Ad–e) is shown. Normal figures showed either one mass in metaphase or two masses in anaphase. (C) Quantitation of spindle defects observed in metaphase and anaphase oocytes. Defective, multipolar, and occasionally monopolar spindles are observed in *gwl^Sr18^/Df* oocytes (as in Ad–e). 149 *Oregon R* oocytes and 142 *gwl^Sr18^/Df* oocytes were scored in these experiments (B and C). (D and E) Rescue of *gwl^Sr18^/Df* female fertility with the expression of *UASp-gwl-long* (D) or *UASp-gwl-short* (E) driven from the *Mat* α*-Tub-Gal4* driver (in two separate experiments). The average numbers of adult progeny per female from 6 d (D) or 7 d (E) of egg laying by females of the indicated genotypes are shown (± standard error of the mean). See Text S1 for details. Females of the genotypes used in (E) were also tested for meiotic chromosome nondisjunction by crossing to *Y^S^X^.^Y^L^, v f B/0; C(4)RM, ci ey^R^/0* males. Numbers of (nullo-4, diplo-X+nullo-X)/total progeny are, from top to bottom: (0, 0)/432; (2, 0)/671; no progeny; (0, 0)/646; (0, 0)/397; and (2, 0)/294.

Gwl accumulation in the oocyte led us to ask whether *gwl^Sr18^*/*Df* oocytes encounter problems during meiosis per se. To examine female meiosis I, we performed immunostaining (for α-tubulin and DNA) in inactivated vitellarial eggs at stages 13 and 14. Wild-type oocytes normally arrest in metaphase I with the larger chromosomes with chiasmata compacted into a single mass at the metaphase plate, while the small fourth chromosomes have moved halfway to the poles as the result of distributive segregation (see *WT* control in Figure 8Aa and [18,19]). We scored the number of DNA masses (ignoring the tiny fourth chromosome) in *WT* and *gwl^Sr18^*/*Df* oocytes. In contrast to the *WT* oocytes, which mostly have only one DNA mass at the metaphase plate, 78% of *gwl^Sr18^*/*Df* oocytes have widely scattered chromosomes, and the number of chromosome masses varies from two to 12 (Figure 8B). Complete homologue separation would give six large chromatin masses; about a quarter of the mutant meioses have more masses than that. Multiple chromatin masses might be caused by reduced meiotic exchange, or precocious loss of sister-chromatid cohesion, or broken chromosomes resulting possibly from faulty meiotic exchange. If meiosis I lacks chiasmata then metaphase I arrest does not occur [18,19], although in wild type anaphase I and II proceed precociously but normally; although some anaphase movements probably occur precociously in *gwl^Sr18^/Df*, they are certainly not normal, so simple reduced exchange does not explain this phenotype though it could be compounded with other problems. A few of the meiotic spindles are aberrantly shaped (Figure 8C; 8% of the total oocytes), but all of the multipolar spindles contain multiple DNA masses. Since the wild-type spindle is nucleated from the central mass of chromatin in meiosis I, these abnormal spindles probably reflect nucleation of individual “mini-spindles” from the multiple chromosome masses.

The fertility of *gwl^Sr18^*/*Df* females is rescued completely by driving transgenic *UASp-gwl-long* in the maternal germline with *Mat* α*-Tub-Gal4* (Figure 8D, and nearly complete egg hatch for all fertile genotypes shown), demonstrating that the sterility and meiotic defects of *gwl^Sr18^* are due solely to the loss of Gwl-long in ovaries. Similar expression of *UASp-gwl-short* in a separate experiment partially rescues the fertility of *gwl^Sr18^*/*Df* females (Figure 8E). That this rescue is only partial is confirmed by partial egg hatch (around 50%) compared with nearly complete egg hatch in all fertile control genotypes. Gwl-long is more competent than Gwl-short for providing maternal function; that there is no significant increase in nondisjunction (Figure 8, legend) suggests that rescue of meiosis per se by Gwl-short is complete or nearly so.

Our attempts to study early embryogenesis in eggs from *gwl^Sr18^/Df* females have been frustrated by the fact that these eggs collapse on handling; this may be related to the yolk-distribution problem. Very gently handled single dechorionated early embryos have all failed to reach cellular blastoderm (our unpublished observations).

### Gwl Is Required for Sister Chromatid Cohesion in Oocytes

**Figure 9:**
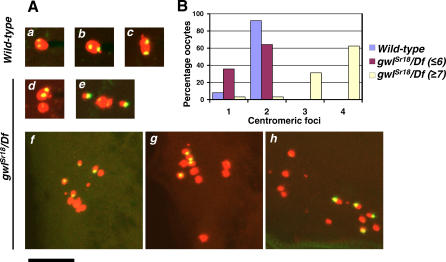
Gwl Is Required for Sister Chromatid Cohesion in Female Meiosis (A) Confocal images of meiotic figures of single oocytes stained for DNA (red) and showing FISH signal for satellite DNA near the centromere of the X chromosome (large green foci). In wild-type oocytes (a–c), one or two large foci are observed, representing the two homologous pairs of centromeres without tension in prophase (one focus) or under tension on the chiasmate bivalent in prometaphase or metaphase I (two foci). Up to four large foci are observed in *gwl^Sr18^/Df* oocytes (d–h), indicating the absence of sister chromatid cohesion. Panels d and e show examples of figures with six or fewer DNA masses; panels f–h show examples of figures with more than six DNA masses. Scale bar is 10 μm. (B) Quantitation of the number of centromeric FISH foci relative to the number of DNA masses in wild-type and *gwl^Sr18^/Df* oocytes (as in [A]). In *gwl^Sr18^/Df* oocytes, the presence of more than two centromeric FISH signals strongly correlates with the presence of more than six large DNA masses, consistent with the loss of sister chromatid cohesion. Numbers of oocytes scored are 75 for wild type, 98 for *gwl^Sr18^/Df* with six or fewer DNA masses, and 32 for *gwl^Sr18^/Df* with seven or more DNA masses.

The presence of more than six DNA masses in *gwl^Sr18^/Df* oocytes could be caused by the absence of sister chromatid cohesion or by DNA damage causing chromosome fragmentation. To distinguish between these two possibilities, we performed fluorescence in situ hybridization (FISH), probing for satellite DNA near the centromere of the X chromosome. In wild type, we observed that 92% of oocytes have two large foci, corresponding to the two homologous pairs of sister centromeres that are under tension in prometaphase and metaphase I while held together as chiasmate chromosomes (Figure 9Aa–c and 9B). In *gwl^Sr18^/Df* oocytes, we often observed three or four centromeric foci, indicating the presence of separated sister chromatids (Figure 9Af–h). This was observed in 94% of mutant oocytes with more than six DNA masses (31% with three foci and 63% with four foci), while only 6% showed one or two foci (Figure 9Ad–e and 9B). We never observed more than four centromeric foci. Moreover, three or four centromeric foci were only found in oocytes that had more than six DNA masses. The DNA masses showing FISH foci were of similar size and shape, which is expected for homologous sister chromatids (Figure 9Af–h). These results indicate the absence of sister chromatid cohesion in some *gwl^Sr18^/Df* oocytes. Therefore, Gwl is required for sister chromatid cohesion in female meiosis I. Indeed, this may be the major, or sole, defect, with the various spindle abnormalities reflecting either successive stages or variability in degree of physical separation at the onset of spindle formation.

## Discussion

The Gwl kinase seems to have multiple roles in progression through mitosis and meiosis. The phenotypes shown by *gwl* mutants differ at different stages of development reflecting both the nature of different alleles and the variety of ways in which the cell cycle is regulated in *Drosophila*. Indeed it is these different modes of cell cycle regulation as development proceeds that allow us to tackle Gwl's multiple functions in cell division.

### Gwl Antagonizes Polo

Our starting point was the strong genetic interaction between the *Scant* mutation and *polo* mutations; heterozygous females lay embryos that die, presumably as a consequence of mitotic failure whose first observed defect is that a single centrosome moves away from the nucleus before nuclear envelope breakdown in all cases examined. This centrosome misbehavior is probably the primary defect; developmental failure probably results from secondary defects of abnormal spindles. It will be interesting to reexamine the phenotype of other maternal-effect mutants showing free centrosomes to see if they disassociate from the nuclear envelope in the same manner. It will also be interesting to find out whether the detaching centrosome always contains either the older or the younger centriole, since they may harbor different amounts of biochemical factors in their pericentriolar material. If so, the history of the centrosome determines its vulnerability to detachment when the Gwl/Polo balance is compromised. The *Scant-polo* genetic interaction is moderately specific since a screen for mutants reverting the maternal-effect embryonic lethality generated only one third-site interactor among two independent *polo* ^+^ duplication events plus two revertants of the *Scant* allele itself. *Scant* encodes a Gwl kinase with a K97M substitution that results in hyperactivity in vitro (albeit with altered specificity on the artificial substrates tested); a wild-type transgene with just this amino acid mutated interacts dominantly with *polo* mutants, so this amino acid substitution is *Scant*. Moreover, mothers overexpressing wild-type Gwl kinase in the presence of reduced Polo kinase function produce embryos with the same kinds of defects as *Scant*. Therefore, the increased activity of Gwl-K97M and not its altered substrate specificity is responsible for the functional interaction between *Scant* and *polo*. It follows that a balance between Gwl and Polo activities in embryos is crucial, but because there does not seem to be any such interaction in cell cycles at later stages (in proliferating larval, pupal, or adult tissues), since *polo^11^ +/+ Scant* itself has normal viability, the balance appears particularly important for these early embryonic cell cycles. The syncytial nuclear division cycles are unusual in that they comprise rapidly alternating cycles of S phase and M phase without intervening gap (either G1 or G2) phases. A G2 phase is only introduced following cellularization when String (the Cdc25 dual-specificity phosphatase that activates Cdk1) is degraded; its expression then comes under transcriptional regulation in a spatio-temporally defined pattern. The critical balance of Polo and Gwl kinase activities in the syncytium may reflect the absence of a G2 state; mitotic proteins are held on continual standby, awaiting their use in the next cycle, rather than being degraded and resynthesized each cell cycle as is the case for cycles with a G2. Alternatively, centrosome detachment may be frequent in other tissues of *polo-Scant* flies where it may be better tolerated. However, we consider this unlikely because we observe only normal centrosome positioning in *polo-Scant* testes (unpublished data).

This antagonism between Polo and Gwl was not predicted from studies of these enzymes in *Xenopus* cell-free systems, which have been used to model the entry into mitosis from G2 through the activation of the Cdc25 phosphatase. There the evidence indicates that both Gwl and Plk1 kinases participate in the autoregulatory loop that activates the Cdk1/cyclin B MPF kinase complex [5,12]. This apparent cooperation of the two kinases in this process suggests that the *Xenopus* cell-free system may be assessing a different aspect of cell cycle progression than our in vivo studies on the syncytial cycles of *Drosophila* embryos. The apparent differences in results may also reflect the different aspects of mitosis under study; activation of Cdk1 in one system and the integrity of the mitotic apparatus in the other. We have few clues about the directionality of the antagonism we observe for Polo and Gwl function in fly embryos or whether it involves direct interactions between the two protein kinases. Yu et al. [12] observed that xPlk1 is capable of phosphorylating xGwl, but they did not observe changes in xGwl activity or a synergistic effect in combination with cyclin B-Cdc2-mediated phosphorylation. However, any inhibitory effect of Polo kinase on Gwl need not be mediated through regulation of kinase activity but could also occur by regulating Gwl's localization or stability. In this case, reduced dosage of Polo in the fly embryo might provide only a subthreshold activity, insufficient for the efficient downregulation of the hyperactive Gwl kinase encoded by *gwl^Scant^*. That Gwl needs to be downregulated is suggested by its subcellular localization in mitosis. Gwl is enriched in the nucleus in interphase, but it is excluded from the nucleus during prophase, before nuclear envelope breakdown. This could occur through active nuclear export or through degradation. In favor of the latter, we observe that Gwl is ubiquitinated (our unpublished observations).

Gwl could also inhibit the function of Polo. Our in vitro experiments suggest that Gwl does not readily phosphorylate Polo (unpublished observations), but it is also possible that phosphorylation of an intermediate substrate by Gwl mediates the hypothetical inhibitory effect. Since *Scant* causes a decrease in female fertility that is stronger in stronger *polo* mutant alleles, and the meiotic divisions occur in the embryonic cytoplasm, it is possible that *Scant* lowers Polo's activity during meiosis. Scott Hawley and colleagues have recently observed a functional interaction between *polo* and *matrimony* (*mtrm*); heterozygous *mtrm/+* females have an elevated frequency of chromosome nondisjunction in meiosis, and this is suppressed by lowering the *polo* dosage [20]. Therefore, if *Scant* acts to lower Polo's activity in female meiosis, then *Scant* might suppress the increased level of nondisjunction in *mtrm* heterozygotes. Indeed it does (Tables S1-4), though *Scant* needs to be homozygous for the suppression to be detectable, so the possibility that this suppression reflects homozygosity of some closely linked third player rather than *Scant* itself cannot be eliminated (see Text S2). Furthermore, even in *Scant/Scant*, the suppression of *mtrm/+* is much weaker than halving the dosage of *polo^+^* directly. This is consistent with the effects of *Scant* on fertility; homozygous *Scant* in a homozygous *polo^+^* background is much more fertile than *polo^11^ Scant/+ +*, so regardless of how *Scant* acts to reduce the functional level of *polo^+^*, one copy of *Scant* does not reduce the activity of one copy of *polo^+^* completely. Nevertheless, the *Scant-mtrm* interaction result strongly suggests that the *polo-Scant* (and probably *polo-gwl*) interaction occurs during female meiosis as well as embryonic mitosis, and the unexpected *Scant-mtrm* interaction lowering female fertility implies a role for *mtrm* in embryogenesis.

That Gwl downregulates Polo's function in the embryo is also suggested by the cellular phenotype, which is in line with the known functions of Polo at the centrosome. Moreover, the *Scant* phenotype is increased by the severity of the *polo* mutant. Occasional displacement of centrosomes early in mitosis is seen in syncytial embryos derived from heterozygous *polo* mutant females themselves (Figure S2), and Polo promotes centrosome separation, maturation, and integrity. In *Drosophila*, Polo phosphorylates Asp and together they promote the recruitment of γ-tubulin to the centrosome [21]. In mammalian cells, Plk1 phosphorylates Nlp, triggering its dissociation from the centrosome and recruitment of several factors [22–24]. Plk1 also phosphorylates Kizuna, which is required to preserve centrosome cohesion [25]. The detached centrosomes observed in the *polo-Scant*-derived embryos do not show a reduction in γ-tubulin staining, and astral microtubules nucleate normally. Similar centrosome detachment was observed in *Scant/+* and *Scant/Scant*-derived embryos, albeit with much lower frequencies. Therefore, it seems likely that the partial loss of Polo activity and the gain of Gwl activity both weaken centrosome function in a similar fashion; this is consistent with the mutually antagonistic interaction between *polo* and *Scant* mutations. Furthermore, we note that centrosome detachment occurs before nuclear envelope breakdown (Figure 3), a time when both Polo and Gwl are enriched around the nuclear envelope in syncytial embryos ([26] and Figure S7). This suggests that coordination between the centrosome, microtubules, and the nuclear envelope before nuclear envelope breakdown is sensitive to the balance between Polo and Gwl. Gwl (or cyclin B-Cdk1, which it activates in frog extracts) may share substrates with Polo and regulate them antagonistically in early mitosis.

Centrosome loss can also occur in response to DNA damage, allowing damaged nuclei to fall into the interior of the syncytial embryo and be discarded from the developing fly [16]. This response depends on Mnk/Chk2 [17]. The centrosome detachment observed in *polo-Scant*-derived embryos does not depend on Chk2 (Figure S3) and thus seems to arise from a more direct effect on the centrosome–nuclear envelope association.

### Gwl Controls Chromosome Dynamics in Mitosis

Our hypomorphic *gwl* mutants do not appear to impact directly upon centrosome behavior in the more conventional cell cycles of the larval central nervous system. Previously, Yu and colleagues reported a long delay in late G2 to anaphase in *gwl* mutant neuroblasts in addition to chromatin condensation defects [13] and have suggested that these defects, particularly undercondensation of chromatin, could all be attributed to the function of Gwl in activating cyclin B-Cdk1, although no direct substrate of Gwl is known [12,13]. However, the prevalence of condensation defects and anaphase bridges that we observed in *gwl* mutant neuroblasts, together with the nuclear localization of Gwl in interphase, suggests to us that Gwl may act directly at the chromosome level. The anaphase bridges observed could be a consequence of tangled chromatids or dicentric chromosomes resulting from telomere fusion or other aberrant DNA damage repair events. When Gwl is depleted from cultured cells, they delay at the spindle assembly checkpoint with high levels of cyclin B and checkpoint proteins at kinetochores. The chromosomes of these cells are scattered upon an elongated spindle as though they have defects in kinetochore function. Since metaphase cells with highly condensed chromosomes accumulate in the larval CNS of *gwl* mutants, prolonged checkpoint arrest probably also occurs here. However, the polyploid cells seen in the null mutant indicate that cells can slip past the checkpoint without segregating their chromosomes; since this also happens in wild-type neuroblasts in the presence of colchicine, polyploidy is probably not a direct consequence of Gwl failure.

### Gwl Is Needed for Sister Chromatid Cohesion in Meiosis

A major role for Gwl kinase in regulating aspects of chromosome behavior is also suggested by the meiotic phenotype seen in *gwl^Sr18^/Df* females. *gwl^Sr18^* disrupts the only form of Gwl expressed during vitellogenesis without disrupting the second mitotic isoform of Gwl. Therefore *gwl^Sr18^* provides a unique opportunity to study how loss of Gwl kinase affects vitellogenesis and meiosis. Although *gwl^Sr18^/Df* ovaries develop normally, yolk distribution is abnormal in stages 13–14, females are sterile, and the severe meiotic defects include scattered chromosomes with separated chromatids and elongated spindles.

Scattered chromosomes could result from a number of problems. One possibility is that Gwl is required for proper meiotic recombination; if so, the multiple DNA masses observed could correspond to chromosome fragments resulting from failure to complete chromatid exchange and to repair double-strand breaks. This would also lead to failure to arrest at metaphase I because bivalents would not be held together by chiasmata [18,19]. This is unlikely for several reasons. First, if the masses were fragmented chromosomes, they should vary widely in size; they do not. Second, Gwl accumulates in the oocyte nucleus and the nuclei of the nurse cells directly connected to the oocyte at (but not before) stage 8, which is much later than the time of meiotic recombination. However, we note that if Gwl is involved in meiotic recombination, the tiny amounts of it present in pachytene (germarial) nuclei could be below the detection limit of our antibody. Third, two of the five nuclei that accumulate Gwl never entered pachytene. Finally, our FISH data (Figure 9) prove that chromatid cohesion fails in *gwl^Sr18^* oocytes, and this is sufficient to account for the scattering of DNA masses observed (Figure 8). The number of DNA masses was often higher than six, the maximum expected number for disassociated bivalent chromosomes, disregarding the tiny fourth chromosomes. Therefore, Gwl is required for sister chromatid cohesion in meiosis I. In the absence of Gwl-long, the premature loss of (or failure to establish) arm cohesion would lead to the release of chiasmata if indeed any are formed.

However, neither of these defects alone is expected to lead to complete female sterility. For example, mutants in *c(3)G* prevent all meiotic recombination but are still partially fertile [27]. Mutants in *ord* do not keep sister chromatid cohesion yet show only a partial loss of female fertility [28–30]. While the dissolution of sister chromatid cohesion in *ord* leads to progression through metaphase I into meiosis II, we have seen no normal meiosis II figures in *gwl^Sr18^* oocytes, though it is possible that the elongated bipolar spindles represent attempts to do meiosis II after a failed anaphase I. Thus, the absence of Gwl-long in meiosis does not lead to a simple lack of meiotic recombination nor does it lead only to a premature dissolution of cohesion. The lack of Gwl could lead to a combination of both defects or to yet some other kind of defect that leads to full female sterility. Even if the occasional meiosis succeeds, it is very likely that these embryos would fail to develop because maternal Gwl-long is expected to be required for early embryonic mitoses; indeed, these embryos fail to reach cellular blastoderm**.**

Most female meioses in *gwl^Sr18^* have scattered chromosomes on a single elongated spindle; we think that the minority (8%) that have multiple bundles of spindle are just the extreme of this scattering, since microtubules are nucleated by the chromatin in the acentriolar oocyte [31]. Failure of karyosome formation might cause this scattering; however, oocytes of earlier stages do at least often form a single karyosome (unpublished data). Mutants that affect the spindle directly such as those affecting the microtubule-associated protein Msps show more spindle defects than chromosome scattering [32]. A mutant disrupting the female germline-specific Cdk1-adaptor Cks30A disrupts the integrity of meiotic spindles in addition to showing chromosome alignment defects, but in this case the chromosome scattering observed is much more modest than that in the *gwl^Sr18^* mutant [33].

How does Gwl regulate sister chromatid cohesion? Our results suggest that Gwl antagonizes Polo, which is known to negatively regulate sister chromatid cohesion. It is therefore possible that the absence of Gwl during meiosis results in excessive and/or premature Polo activity, leading to premature loss of sister chromatid cohesion. In budding yeast, Polo (Cdc5) promotes the cleavage of the cohesin Scc1 by direct phosphorylation [34]. In meiosis, sister chromatid cohesion is protected at centromeres until anaphase II by Mei-S332 in *Drosophila* (Shugoshin in other organisms; reviewed by [35]). Indeed, *mei-S332* mutants show premature sister separation in meiosis I [36]. In budding yeast, Shugoshin prevents cleavage of the cohesin Rec8, which replaces Scc1 in meiosis (reviewed by [35,37]), and Cdc5 is required in meiosis for cleavage of Rec8 [38,39]. In *Drosophila*, Polo also negatively regulates Mei-S332 activity and localization [9]. Thus, the lack of Gwl in meiosis could lead to premature activity of Polo, which could negatively regulate Mei-S332 and lead to precocious sister separation in meiosis I. We have examined Mei-S332′s localization in *gwl^Sr18^* hemizygous oocytes and found that Mei-S332 was largely properly localized to centromeres (unpublished data). However, Mei-S332 can be inactivated even when it remains localized at centromeres [9]. Therefore, we cannot rule out the possibility that Mei-S332 is being negatively regulated in the absence of Gwl. Alternatively, Gwl could promote sister chromatid cohesion by directly phosphorylating effectors of cohesion. Gwl-long is better than Gwl-short at performing a maternal function and we suspect that Gwl-long will be a better kinase for a yet unknown maternal substrate.

### Gwl Has Multiple Roles in Cell Division

In conclusion, it appears that Gwl, in common with the other major mitotic protein kinases, has multiple roles in mitotic and meiotic progression. These have been revealed through a series of *gwl* alleles that exhibit different characteristics and reveal aspects of Gwl kinase function in the different types of cell cycle during *Drosophila* development. A gain-of-function allele of *gwl* reveals a requirement for coordinate activity of the Gwl and Polo kinases in the rapidly oscillating M and S phase cycles of early embryos. Partial and total loss of Gwl function leads to frequent chromosome condensation defects and anaphase bridge formation in the conventional division cycles of cells in the larval CNS. Finally, loss of Gwl function in the female germline leads to severe meiotic abnormalities including loss of sister chromatid cohesion. It will be of interest to identify potential binding partners of the Gwl protein kinase both in interphase, when it is present predominantly in the nucleus, and in mitosis, when it moves out to the cytoplasm. This may in turn facilitate the identification of its substrates; this is crucial for understanding exactly how it regulates these various aspects of cell division.

## Materials and Methods

### Genetics.

The isolation of the *Scant* mutation, the generation of Scant revertant alleles by X-ray mutagenesis, and the mapping of *Scant* revertants and of *Scant* using complementation tests, imprecise excision of *P*-elements, and sequencing are all described in detail in Text S1. Details of the rescue experiments are also provided in that section.

### Plasmids.

Plasmids were constructed following the Gateway technology. The *gwl-long* cDNA was PCR-amplified from cDNA LD35132 (Flybase sequence) with primers including attB BP recombination sites at both ends, permitting cloning into the *pDONR221*λ entry vector to generate *pDONR-gwl-long* (with STOP codon or without it for C-terminal tagging). The *gwl-short* cDNA was cloned using the same primers from total cDNA from S2 cells to generate *pDONR-gwl-short. pDONR-gwl-long-K97M* and -*K87R* (kinase dead) were made by PCR-based point mutagenesis. Entry clones were sequenced; *gwl-long* was found to match the Flybase sequence, the K97M and K87R mutants differed from *gwl-long* by those single mutations only, and *gwl-short* was found to lack exon 4 exactly. For transgenic lines, *pUASp-gwl-long*, *pUASp-gwl-long-K97M*, *pUASp-gwl-long-EGFP*, *pUASp-gwl-long-K97M-EGFP*, and *pUASp-gwl-short* were made by LR recombination of the entry clones into a *pUASp* or *pUASp-C-termEGFP* Gateway destination vector. For cell lines, *pAC5-myc-gwl*, *pAC5-myc-gwl-K97M*, and *pAC5-myc-gwl-K87R* were made by LR recombination into *pAC5-N-termMyc*.

### Generation of Gwl antibodies.

The sequence encoding Gwl residues 352–846 was PCRed from cDNA LD35132 and was inserted into *pET23b* vector to produce the protein in fusion with a C-terminal His(6) tag in Escherichia coli after IPTG induction. The protein was purified under denaturating conditions (BugBuster, Novagen). Antibodies were raised after injection of the purified protein in rabbit (Harlan Sera-Lab).

### Immunofluorescence.

Embryos were collected every 2–3 h, dechorionated by incubation in 50% bleach for 2 min, rinsed in water, and incubated with agitation for 5 min in permeabilizing-fixative solution (55% heptane, 15% formaldehyde, PBS). The vitelline membranes were then removed by repeated washes in methanol, and embryos were rehydrated initially in methanol/PBS (1:1) and then in PBS containing 0.1% Tween 20 (PBT). For immunofluorescence, blocking was performed with PBT containing 1% BSA (PBTB) for at least 30 min. Antibodies were as follows (diluted in PBTB): monoclonal rat anti-α-tubulin YL1/2 (diluted 1:20); mouse monoclonal anti-γ-tubulin GTU88 (Sigma T6557, diluted 1:50); anti-rat-FITC (Sigma F-6258, diluted 1:100); and anti-mouse-Rhodamine Red (Jackson Immunochemicals 715-295-151, diluted 1:100). Embryos were incubated with primary antibodies overnight at 4 °C, rinsed in PBT, and washed repeatedly in PBT at RT, incubated with secondary antibodies for 2 h at RT, rinsed in PBT, and washed repeatedly in PBT. To stain the DNA, embryos were washed with PBS, incubated with a 1:1,000 solution of Toto-3 dye in PBS, and washed in PBS. Immunofluorescence in S2 cells was performed essentially as described [11,40]. To visualize female meiosis, ovaries were dissected and fixed for immunofluorescence essentially as described [41] except that BSA (1%) was used as the blocking agent and DNA was stained with propidium iodide.

### Cell culture.

Dmel cells were maintained in SFM medium supplemented with 1 mM Glutamine, penicillin, and streptomycin. S2 cells were maintained in SFM supplemented with 10% calf serum, penicillin, and streptomycin.

### Immunoprecipitations and kinase assays.

Cells (around 1 × 10^8^) stably expressing Myc-tagged proteins were lysed in 1 ml of extraction buffer (50 mM Tris-Cl [pH 7.5], 110 mM NaCl, 50 mM β-glycerophosphate, 5 mM MgCl_2_, 1 mM Na_3_VO_4_, 0.1% NP-40, Complete Protease Inhibitors [Roche] at its recommended dilution, 0.1 mg/ml DN-EP DNase [Sigma]) with four cycles of freeze-thaw on dry ice and cold water. Lysates were centrifugated at 14 krpm on a tabletop centrifuge. A total of 25 μl of 9E10 anti-Myc antibody (Santa Cruz) and 25 μl of Protein G-conjugated Dynabeads (Invitrogen) were added to the clarified supernatants, which were then incubated with agitation at 4 °C for 2 h. Beads were washed four times 5 min with 1 ml of extraction buffer without DNase and once with kinase buffer (20 mM K-HEPES [pH 7.4], 2 mM MgCl_2_, 1 mM DTT) and resuspended in 30 μl of kinase buffer. Kinase assays were performed on beads in kinase buffer using 1 μg of either Histone H1 (Roche), Histone H3 (Roche), Casein (Sigma), or Myelin Basic Protein (Sigma) in the presence of 1 mM ATP and 10 μCi of 32-P-gamma-ATP in a total volume of 20 μl. Reaction mixtures were incubated for 20 min at 30 °C, with occasional agitation, and reactions were terminated by the addition of 2× Laemmli SDS-PAGE sample buffer. Samples were then resolved by SDS-PAGE, transferred to nitrocellulose, and analyzed by autoradiography.

### Microscopy and flow cytometry.

Fixed embryos and ovaries were examined on a Nikon Optiphot fluorescence microscope equipped with BioRad MRC1024 confocal scanner and software. Fixed S2 cells were visualized on a Zeiss Axiovert 200 fluorescence microscope with Metamorph software. Videos of embryos were made on a Zeiss Axiovert 200 fluorescence microscope equipped with the PerkinElmer UltraVIEW confocal scanner and software. Flow cytometry was performed as described [11].

### Brain squash and orcein staining.

Brains were dissected from third-instar larvae in 0.7% NaCl, fixed in a solution of methanol, acetate, and water (11:11:2) for 1 min, transferred to 8 μl of orcein dye on a coverslip, stained for 1–2 min, covered with a glass slide, flipped over, and squashed manually. Only fields with abundant cells were counted.

### FISH.

FISH for satellite DNA near the centromere of the X chromosome (1.688 g/cm^3^ satellite on the left arm) was performed essentially as described [29,42]. DNA was stained with propidium iodide.

## Supporting Information

Figure S1 Second Round Generation of *Scant Revertant* Alleles (A) Females heterozygous for both the *Scant* mutation and a loss-of-function *polo* mutation (such as *polo^11^*, here in *cis*) lay embryos that die during development.
(B) Second genetic screen to generate Scant revertant (*Sr*) alleles. Unlike in the first round of mutagenesis in which *trans*-heterozygous males were mutagenized (Figure 1B), here a *polo^11^ Scant* recombinant chromosome made from the fertile *polo^11^ Dp(polo^+^) Scant* females was mutagenized and tested for fertility over a third-chromosome balancer. In this screen, all ten recessive mutations recovered were allelic to each other and to *gwl*. See Results and Text S1 sections for details.
(40 KB PPT)

Figure S2 Centrosome Disassociation Is Occasionally Observed in Embryos Derived from Females Heterozygous for *polo* or *Scant* Singly Embryos were collected for 2–3 h, dechorionated, and fixed for immunofluorescence. Stainings are α-tubulin (green), γ-tubulin (red), and DNA (blue). The scale bar represents 10 μm.
(312 KB PPT)

Figure S3 The Centrosome Disassociation of *polo-Scant-*Derived Embryos Is Still Observed in the Absence of Mnk/Chk2 Embryos were collected for 2–3 h, dechorionated, and fixed for immunofluorescence. Stainings are α-tubulin (green), γ-tubulin (red), and DNA (blue). Two images are shown to illustrate the range of phenotypes observed for this genotype. Centrosome disassociation was observed in all embryos of this genotype. The aberrant DNA masses and microtubule bundling sometimes seen in *mnk/mnk;* *polo^11^ Scant/+ +*-derived embryos (B) are characteristic of the *mnk* mutation alone (see [3] in Text S3). The *mnk* allele used is the null *lokP6* (provided by W. Theurkauf), which we verified by PCR.
(279 KB PPT)

Figure S4 Gwl Depletion by RNAi in S2 Cells and Associated Phenotypes (A) Western blot for Gwl after RNAi in S2 cells using double-stranded RNA targeting total Gwl (double-stranded RNA targeting exon 1) or Gwl-long only (double-stranded RNA targeting exon 4); GFP double-stranded RNA is the control. * indicates a cross-reacting band serving as a loading control.
(B) Chromosomes are scattered along an elongated spindle, and BubR1 is present at kinetochores in total Gwl-depleted S2 cells undergoing abnormal mitosis. The control cell is in early anaphase.
(C) Cyclin B levels remain high in Gwl-depleted S2 cells undergoing abnormal mitosis. Depletion of Gwl-long only had no detectable effect. Scale bars are 10 μm.
(2.8 MB PPT)

Figure S5 Examples of Somatic Defects Observed in *gwl* Mutant Flies Rough eyes and ragged wings observed in *gwl^Sr3^/gwl^Sr6^* mutant flies are rescued by *UASp-gwl-long* driven by *Actin 5C-Gal4*.
(1.2 MB PPT)

Figure S6 Quantitation of Defects Observed in *gwl* Mutant Neuroblasts of Additional Genotypes (to Be Compared with the Data in Figure 6) Quantitation of defects observed in larval neuroblasts following orcein DNA staining (as in Figure 6) for *gwl^Sr3^/gwl^6^* and *gwl^Sr6^/gwl^6a^* (both partial loss of function genotypes). Mitotic cells were recognized by their condensed chromatin. The M/A ratio corresponds to the number of prometaphase and metaphase cells over the number of anaphase and telophase cells (F, number of fields scored). % condensation defects, number of mitotic cells with under- or overcondensation/total number of mitotic cells (*n*); % chromatin bridging, number of bridged anaphases/total number of anaphases (*n*); % polyploidy, number of polyploid mitotic cells/total number of mitotic cells (*n*).
(149 KB PPT)

Figure S7 The Localization of Gwl Is Cell Cycle-Regulated in Embryos The strong genetic interaction between *Scant* and *polo* mutations suggests that Gwl and Polo activities have to be coordinated in the cell division cycle. We used time-lapse to examine the cell cycle localization of Gwl-EGFP in embryos, using *Mat* α*-Tub Gal4* to drive expression in the germline. In syncytial embryos (left), Gwl-EGFP is concentrated in the nucleus in interphase and concentrated around the nucleus in early mitosis, presumably before nuclear envelope breakdown (the arrow indicates the direction of the mitotic wave). In cellularized blastoderm, Gwl-EGFP is also concentrated in the nucleus in interphase (T_0_), but then becomes concentrated around the nuclear envelope and largely disappears from the nucleus (below cytoplasmic levels) shortly before nuclear division (9–12 min). It then becomes more evenly distributed throughout the cell, presumably after the nuclear envelope has become broken or fenestrated (12–18 min). Gwl-EGFP becomes clearly nuclear again at the end of nuclear division (21–23 min). Gwl-K97M-EGFP behaves identically to Gwl-EGFP (unpublished data), ruling out the possibility that the genetic effect of *Scant* (on *polo* mutants) could be due to abnormal subcellular localization of Gwl caused by the K97M substitution. This localization pattern suggests that Gwl is being actively imported into the nucleus in interphase and either degraded in the nucleus or exported to the cytoplasm in early mitosis.
(2.6 MB PPT)

Table S1 Progeny from Females with Normal X Chromosomes and the Indicated Third-Chromosome Genotype Crossed to *Y^S^X.Y^L^, v f B/0; C(4)RM, ci ey^R^/0* Males Parental crosses were *FM7/+; sr Scant* females x *y w/Y; mtrm^13^/TM3_13_* or *y w/y^+^Y; mtrm^126^/TM3_126_* males and *FM7/Df(1)p116D, w* females by both genotypes of *mtrm* males. *y w/w Df* females from the latter cross produce only half the expected number of regular males, since the deficiency is recessive lethal; for these progeny, the number of regular males has been doubled (parentheses) before calculating gametic frequencies. Although diplo-*X* ova produce phenotypically distinct progeny here, they have been included in the calculations as though they could not be distinguished from mono-*X* ova, so the frequencies here will be comparable to those in Table S4. Tests set up on different days are presented separately.
(46 KB DOC)

Table S2 Progeny from *FM7/*+ Females with the Indicated Third-Chromosome Genotype Crossed to *Y^S^X.Y^L^, v f B/0* *; C(4)RM, ci ey^R^/0* Males Females tested are sibs of those in Table S1. *FM7/0* males are nearly always lethal.
(31 KB DOC)

Table S3 Progeny from Females with Normal X Chromosomes and the Indicated Third-Chromosome Genotype Crossed to *Y^S^X.Y^L^, v f B/0; C(4)RM, ci ey^R^/0* Males Parental crosses were *FM7/+; sr Scant* females *x + Scant_f_ / TM6B_f_, mtrm^13^ Scant/TM6B_13_,* or *mtrm^126^ Scant/TM6B_126_* males.
(30 KB DOC)

Table S4 Progeny from FM7/+ Females with the Indicated Third-Chromosome Genotype Crossed to *Y^S^X.Y^L^, v f B/0; C(4)RM, ci ey^R^/0* Males Females tested are sibs of those in Table S3.
(29 KB DOC)

Text S1 Supporting Materials and Methods (49 KB DOC)

Text S2 Supporting Results (27 KB DOC)

Text S3 Supporting References and Accession Numbers (31 KB DOC)

Video S1 Wild-Type (Oregon R) Syncytial Embryos (GFP-β-Tubulin): Control for Video S2
Frames were taken every 20 s. Exposure time was 300 ms. Spacing between images was 0.5 μm.
(8.9 MB AVI)

Video S2 *polo^11^ Scant*/+ + -Derived Syncytial Embryos (GFP-β-Tubulin) Some nuclei undergo an apparently normal mitosis, while for others one centrosome initially disassociates but is eventually recaptured, allowing mitosis to be completed normally. In other nuclei, the free centrosome drifts far away from its spindle. These centrosomes are not recaptured and the mono-centrosomal spindles can eventually fuse with each other or proceed to produce a bipolar spindle and divide. Frames were taken every 20 s. Exposure time was 300 ms. Spacing between images was 0.5 μm.
(8.9 MB AVI)

## Abbreviations

Cdk1: cyclin-dependent kinase 1
FISH: fluorescence in situ hybridization
GFP: green fluorescent protein
Gwl: Greatwall
*mtrm,*: *matrimony;*
RNAi: RNA interference
*Scant,*: *Scott of the Antarctic;*
*Sr,*: *Scant revertant;*
wt: wild type
