# Supplementary material for: Mutations in Drosophila Greatwall/Scant Reveal Its Roles in Mitosis and Meiosis and Interdependence with Polo Kinase
Source: PLoS Genet. 2007 Nov 9;3(11):e200. doi: 10.1371/journal.pgen.0030200 (PMC2065886; doi:10.1371/journal.pgen.0030200)
Supplement: Supplementary file 1 [file pgen.0030200.sg006.pdf]

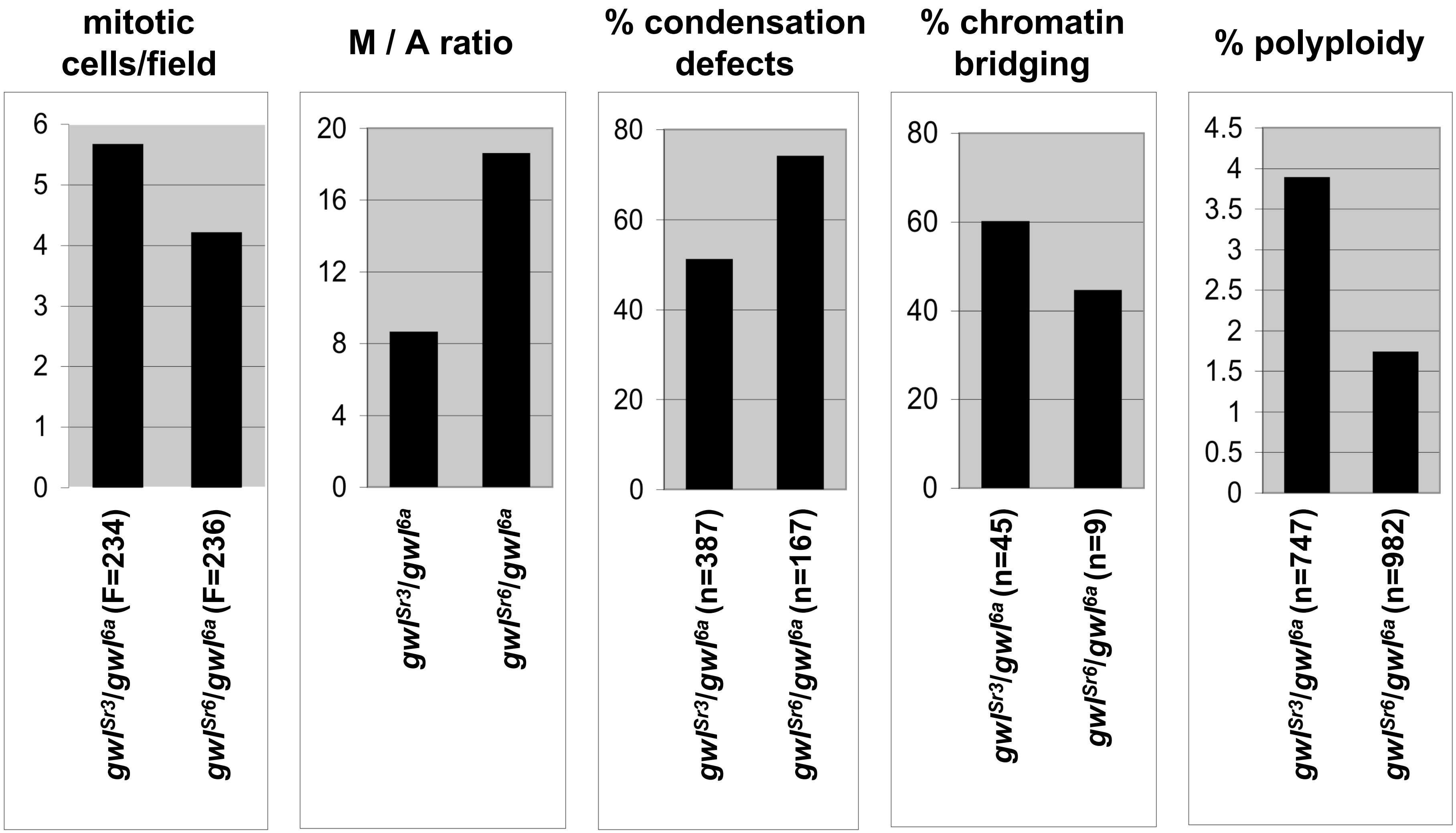
mitotic cells/field
6
5
4
3
2
1
0
gwl^{Sr3}/gwl^{6a} (F=234)
gwl^{Sr6}/gwl^{6a} (F=236)
M / A ratio
20
16
12
8
4
0
gwl^{Sr3}/gwl^{6a}
gwl^{Sr6}/gwl^{6a}
% condensation defects
80
60
40
20
0
gwl^{Sr3}/gwl^{6a} (n=387)
gwl^{Sr6}/gwl^{6a} (n=167)
% chromatin bridging
80
60
40
20
0
gwl^{Sr3}/gwl^{6a} (n=45)
gwl^{Sr6}/gwl^{6a} (n=9)
% polyploidy
4.5
4
3.5
3
2.5
2
1.5
1
0.5
0
gwl^{Sr3}/gwl^{6a} (n=747)
gwl^{Sr6}/gwl^{6a} (n=982)
