# Supplementary material for: Mutations in Drosophila Greatwall/Scant Reveal Its Roles in Mitosis and Meiosis and Interdependence with Polo Kinase
Source: PLoS Genet. 2007 Nov 9;3(11):e200. doi: 10.1371/journal.pgen.0030200 (PMC2065886; doi:10.1371/journal.pgen.0030200)
Supplement: Supplementary file 2 [file pgen.0030200.sd001.doc]

**Supporting Materials and Methods**

***Isolation of the* Scant *mutation:*** *Scant* was originally isolated as a dominant enhancer of *polo* female sterility [1]. The original *Scant* chromosome was recessive female sterile as well, and the original mapping of the *Scant*-bearing chromosome, which placed *Scant* in the middle of 3R (therefore not just another *polo* allele), failed to separate these two phenes [1]; however, a variety of contradictions led us to re-do the genetic mapping, testing each crossover both for recessive female sterility over the original *Scant* chromosome and for dominant enhancement of *polo* female sterility. Of 18 crossovers between *sr (stripe,* 62.0) and *e (ebony,* 70.7), one *sr e^+^* crossover was only the dominant enhancer (this chromosome confers no obvious phenotype when placed over the original *Scant* chromosome or over a deficiency and can easily be maintained in stock as homozygotes) and two *sr^+^ e* crossovers were only the recessive female sterile(s). The latter have been named *nots = notScant* and not studied further; all experiments reported herein have been done with cleaned-up *Scant*-only chromosomes -- which now are all marked with *sr*, only approximately 1.5 map units from *Scant*, allowing it to be followed reliably through crosses.

***Generation of* Scant revertant *alleles and mapping****:* Since *Scant* alone has no obvious phenotype other than its enhancement of *polo*, it is impossible to deficiency-map it; however, the fact that the mutation is dominant suggests that it is hypermorphic or neomorphic, and if so it should be revertible by mutations that disable its gene (which is either still functional despite the dominant change or not vital). We reasoned that such revertants should restore female fertility when placed over *polo* mutations; the few fertile females will select themselves against the background of sterile sisters. Therefore homozygous *sr Scant* males were given 50 Gy of X-rays, crossed to *polo^11^, st pp red e/TM6C* females (*polo^11^* is *In(3L)77B1-3;77E1-2*, detected from a separate experiment asking for mutations making *Scant* a dominant female sterile; since it has one breakpoint in *polo*'s salivary band (77B2) it is likely to be an amorphic allele), and *polo^11^ +/+ Scant** (* is the irradiated chromosome) virgins were crossed in batches of 10 to appropriate males from which positive chromosomes could be balanced. Since this experiment will detect not only hits that reduce the function of *Scant*'s gene but also third-site interactors, and also because the tested females are not balanced for the third so crossing-over should be uninhibited, multiple stocks, each from an individual *e^+^* progeny, were established from each positive culture and each was retested for suppression of *polo* sterility. Five solid suppressing events (*Sr* = *Scant revertant*) were recovered out of the 5492 chromosomes tested, though the background was unexpectedly high; about one in 8 vials (= 80 females) had a few larvae that failed to produce adults and about one vial in 50 produced a few adults that failed on retest. This leakage is temperature-sensitive; it is increased by culture at 18oC relative to the normal 22oC but this effect is too subtle to allow determination of the temperature-sensitive stage.

Two of the positive *Scant revertants*, *Sr3* and *Sr6*, appeared likely to be the desired hits in *Scant*'s gene. They retested as positives, still carried *sr*, and had gained recessive sub-vital mutations which were allelic and which deficiency-mapped just to the right of *sr*, where the dominant mapped. *Sr3* has no cytological defect and its lesion is hypomorphic; homozygotes (on good food in uncrowded cultures) have eclosion delay but eventually the expected 1/3 eclose. These adults do have a range of phenotypes, such as slightly to very reduced rough eyes and incomplete abdominal cuticle, indicative of difficulties in cell-cycle progression. *Sr3/Df* progeny have reduced viability as well as more severe eye and wing phenotypes. *Sr6* has an (unrelated?) cytological defect; it is a tandem duplication for 90EF;92DE. This should mean two copies of *Scant*, which is in the middle of the region duplicated; however, *Sr6* does behave as a simple reduction of function and only one PCR product is recovered for any part of the *gwl* gene. Although its chromosome is homozygous lethal, this is from some second event since it gives escapers over deficiency (with the same phenes as *Sr3*); moreover, *Sr6/Sr3* behaves more like *Sr3/Sr3* than like *Sr3/Df*, indicating that *Sr6* is also a hypomorphic allele, though somewhat more defective than *Sr3*. The frequency of hits in *Scant'*s gene, 2/5492, is well within the range of decrease-of-function hits in other recessive genes (*st, p, e, ca*: 0.9-4.6 x 10^-4^) that were monitored to gauge the effectiveness of the mutagenesis.

Two of the positives are duplications for *polo^+^* -- *Dp(3;3)polo-Sr4,76D5-8;77E3-5* into *83A* and *Dp(3;3)polo-Sr8,76D1;77E1-3*. Though unexpected (X-rays hit mature sperm, only one copy of the genome is present -- but I [ATCC] had left the treated males with their females long enough so that pre-meiotic events could have been recovered), these events both nail the point of the *polo-Scant* interaction (see Results) and provide material for the next experiment.

The final positive is a deficiency in 3L, which identifies a real third-site interactor since independent deficiencies of its region also suppress the sterility of *polo-Scant* females.

This first run worked so well it clearly should be carried forward, now with *polo^11^* and *Scant* in cis for efficiency (Figure S1); *polo^11^, st pp red e/Dp(3;3)polo-Sr4* or *Dp(3;3)polo-Sr8, sr Scant* females were crossed to *ruPrica* males and scarlet striped crossovers were selected -- as, and kept as, males, since the desired *polo^11^ Scant* double-mutant chromosome is female sterile. Two different lines (one each from *Sr4* and *Sr8*) were kept and each was used for X-ray mutagenesis; they behaved identically. Males were again treated with 50 Gy but now they were crossed to *TM6C* females and *polo^11^ Scant/TM6C* daughters were tested for fertility again in batches of 10 (and the X-rayed fathers were removed after 6 days). This run did give positives but most were large deficiencies that removed the *Scant* mutant gene outright; the balancer-heterozygous females also gave much lower levels of leakiness than the first run, suggesting that the deleterious effects of balancer heterozygosity were tipping what should have been partly-fertile positives into cultures too infertile for recovery of the positive chromosome. Late in the run *polo^11^ Scant*/+* + sisters were also tested; leakiness went back up but no positives were recovered from the few such females.

This second run produced 4 cytologically visible deficiencies (all removing 91C5,6, *Scant*'s band), one T(2;3) [56F10-17;91C3-D5] and two invisible deficiencies (detected as deficiencies by PCR) out of 14,789 females tested as well as three cytologically-normal positives, *Sr17, Sr19*, and *Sr18*. *Sr17* and *Sr19* are very similar and indeed could have come from the same father; they have indistinguishable changes in the vicinity of the *Scant* gene. Neither gives a PCR product for the kb 5' of *gwl* (between 71k and 72k, Figure 1); this is unlikely to be a large deletion because attempts to PCR across this region from either side also fail, although the adjacent regions themselves PCR up fine. We suspect the insertion of a natural transposon 5' of *gwl,* down-regulating it. Both are only semi-fertile in the presence of a *polo* allele but fully fertile in a *polo^+^* background, and neither displays any obvious recessive defect except that fertility of *polo^11^ Sr17* or *Sr19/Sr3* females is even more reduced. *Sr18* is fully viable and phenotypically normal in the absence of *polo* but females are completely sterile (lay lots of eggs that do not develop); this same sterility occurs in *Sr18/Sr3* or *Sr18/Sr6* females, so *Sr18* is a hit that disrupts *Scant*'s maternal effect without affecting the somatic effect of its gene.

The various mappings of the recessive *Scant* hits narrowed its recessive subvital to *Df(3R)Exel6181, 91C5;91D5*, bases 14 554 953-14 770 980; the next step was to mobilise *P* inserts in the region to induce imprecise excisions, crossing all progeny either to *Df(3R)Exel6181* and testing lethals for a recessive *Scant* phenotype (both positive and negative results would have been informative) or to *Sr3/Bal* directly. Several spaced insertions were tested; the one that proved fully successful was *gwl^EP515^*, inserted 217 bp 5' of gwl and 12 bp 5' of *CG7718*. Although the original insertion chromosome carries a nearby, unrelated recessive lethal (removed by crossing-over in the positives before further study), the insertion itself over deficiencies for its region is fully viable, phenotypically normal, and fully fertile in both sexes. All of the *P*-chromosome-carrying progeny of seven *P-insert/Dr delta 2-3* males were tested, sorted by father so that pre-meiotic clusters could be identified; seven useful positives were recovered (see below for sequence information). Three independent events (*gwl^2^*, *gwl^3a^*, and *gwl^6a^)* fail to complement *Sr3*; three independent events (*CG7718^1^*, *CG7718^6b^*, and *CG7718^7^)* complement *Sr3* but fail to complement each other; and one event (*gwl^3b^)* fails to complement both. *gwl^2^, gwl^3a^, gwl^6a^,* and gwl*^3b^* have white eyes, having lost the inserted mini-white; the rest retain the lemon eye color of the original insertion line. All of the events that fail to complement *Sr3* not only give (mutant) escapers at the reduced frequency diagnostic of large deficiencies, they also give the occasional escaper as homozygotes or over larger deficiencies, and the lethality is mostly late pupal lethal.

Since the P-hop experiment implies that either *gwl* or *CG7718* is the *Scant* gene, primers were designed for PCR at 1 kb intervals across both genes. All three of the events that uncovered *Sr3* extend distal into *gwl* (= one or more kbs failed to PCR up); all three that complemented *Sr3* extend proximal into or past *CG7718*; and *gwl^3b^* removed sequences both ways. The recessive phenotypes identified by *Sr3* *etc.* are therefore likely to be defects in *gwl*. Of course, *Scant* itself was sequenced first; there are 30 differences within the transcribed sequence between *Scant* and the genome sequence, 25 in introns or silent codons, 5 changing an amino acid when compared with the FlyBase sequence -- K97M, S391G, T395S, P432Q and E846D.

Although what stock presently in the Glover-lab collection might have been *Scant*'s progenitor is lost in the mists of time, the original stocks of several of the other mutations recovered from its mutagenesis are still present; their *gwl^+^* genes were PCRed and sequenced and they do have all the silent and amino acid changes of *Scant* except one: K97M (base 14,573,098 is changed from an A to a T; this changes amino acid 97 from Lys to Met). This suggests that the K97M substitution in *gwl* is the dominant *Scant* mutation that results in the sterility of heterozygous *polo* mutant females. That this is the *Scant* mutation has been confirmed by artificial induction and transformation (see below). Sequencing the *gwl* gene in *Sr3* identified a 27 base pair deletion, removing 9 codons for the residues 156-164 in the N-terminal part of the kinase fold (Figure 5). *Sr6* has an ochre codon in place of lysine 678. Both mutations retain the same five codon differences (compared with the FlyBase *gwl* sequence) as *Scant,* including K97M.

***Rescue of viability and fertility tests:*** To ask whether Gwl-long or Gwl-short could rescue the viability of *gwl^Sr3^/gwl^Sr6^* mutants, *Act5C-Gal4 UASp-gwl-(long or short)/CyO; gwl^Sr6^/MKRS* or *+/CyO; gwl^Sr6^/MKRS* (control) males were crossed to *gwl^Sr3^/TM6C* females*.* *CyO/CyO* and *MKRS/TM6C* are lethal, and *gwl^Sr3^/gwl^Sr6^* is largely inviable on the food used in this experiment (without *gwl* transgenic expression) so the few escapers were excluded from the total progeny number used in the calculations. This leaves 5 (out of 8) classes of progeny. If rescue is complete, we expect the number of *Act5C-Gal4, UASp-gwl-(long or short)/+; gwl^Sr3^/gwl^Sr6^* progeny to be 1/4 of the sum of the remaining 4 progeny classes. Therefore, the number of *Act5C-Gal4, UASp-gwl-(long or short)/+;gw^Sr3^/gwl^Sr6^* progeny flies was divided by 1/4 of the total number of the remaining 4 classes, giving the percentage of rescue. In the control crosses, *UASp-gwl-(long or short)* was replaced by a + chromosome.

To ask whether Gwl-long rescues the fertility of *gwl^Sr18^/Df* mutants, *Mat α-Tub-Gal UASp-gwl-long; Df(3R)Exel6181/MKRS* males were crossed to *gwl^Sr18^/TM6B* females. Three-day-old *Mat α-Tub-Gal4 UASp-gwl-long/+; gwl^Sr18^/Df(3R)Exel6181* females (and their siblings) were tested for fertility individually with 3 *Oregon R* males. Females were allowed to lay eggs for 3 days, then transferred to fresh food for one day, three times (6 days egg laying total). The total number of progeny was scored for 3 to 4 females of each genotype and averaged.

A similar experiment was done to ask whether Gwl-short rescues the fertility of *gwl^Sr18^/Df* mutants. *w^1118^;* *Mat α-Tub-Gal UASp-gwl-short/(If* or *CyO); gwl^Sr18^/TM6B* males were crossed to *w^1118^; If/CyO; Df(3R)Exel6181/TM6B* females. Three-day-old *w^1118^; Mat α-Tub-Gal4 UASp-gwl-short/If; gwl^Sr18^/Df* females (and their siblings) were tested for fertility individually with 3 *Oregon R* males. Females were allowed to lay eggs for 3 days, then transferred to fresh food for one day, two times, and transferred again on fresh food for two days (7 days egg laying total). The total number of progeny was scored for 5 to 9 females of each genotype and averaged.
