# Supplementary material for: Mutations in Drosophila Greatwall/Scant Reveal Its Roles in Mitosis and Meiosis and Interdependence with Polo Kinase
Source: PLoS Genet. 2007 Nov 9;3(11):e200. doi: 10.1371/journal.pgen.0030200 (PMC2065886; doi:10.1371/journal.pgen.0030200)
Supplement: Supplementary file 3 [file pgen.0030200.sd002.doc]

**Supporting Results**

***Scant* weakly suppresses nondisjunction in *matrimony* mutants**

Since reducing the level of *polo^+^* in *mtrm* (*matrimony*) heterozygotes reduces female meiotic nondisjunction [2], and since *Scant* appears to act maternally by reducing the amount of available *polo^+^* in the zygote (this study), it was of interest to see whether heterozygous *Scant* reduced the amount of meiotic *polo^+^* enough to affect X and 4th nondisjunction in *mtrm* heterozygotes. Accordingly, doubly-heterozygous *mtrm* *polo^+^ +/mtrm^+^ polo^+^ Scant* females were produced and crossed singly as virgins to *Y^S^X.Y^L^, In(1)EN, v f B/0; C(4)RM, ci ey^R^/0* males with single-mutant and balancer siblings also test-crossed as controls (Tables S1 and S2). *mtrm* females show the expected rise in X and 4 nondisjunction plus *FM7* (for the X, from ca 15% to 50%); adding one dose of *Scant* has no effect, neither an increase in the absence of *FM7* nor a decrease in its presence. This suggests that *Scant* does not affect *polo^+^* levels during female meiosis, although since weak *polo* alleles heterozygous do not suppress *mtrm*, alternatively one dose of *Scant* may reduce *polo^+^* during meiosis too, just not enough to affect *mtrm*. + *Scant/mtrm* + females are two-thirds as fertile as *mtrm/+*, and adding *FM7* reduces fertility by another half, relative to either + *Scant/mtrm +* or *mtrm/+*.

Although homozygous *Scant* alone has only a mild reduction in female fertility and no increase in nondisjunction, perhaps two doses of *Scant* would reduce *polo^+^* enough to interact with *mtrm* and reduce nondisjunction. Fortunately, an X-ray induced new *scarlet (st)* allele on the *Scant* chromosome was kept; *mtrm^+^* or *mtrm + +/+ st^X^ sr Scant* females were produced, crossed to *ruPrica/TM6C* males, and individual st^+^ sr Pr males were crossed to */TM6B* to balance the crossovers; subsequently, each was tested for *Scant* by female sterility over *polo^11^* and for *mtrm* by homozygous female sterility, if viable. *mtrm Scant/+ Scant* females do show around a two-fold reduction (Tables S3 and S4); since only nullo-X gametes can be recognised from these *FM7/+* females, all crosses are presented with nullo-X only for comparison; and only nullo-4 gametes can be recognised since the maternal fours are unmarked. *+/+*; *mtrm/+* females gave 5-14% nullo-X, 6-14% nullo-4; plus homozygous *Scant* these drop to 4-5% and 4-5%. *FM7/+*; *mtrm/+* females gave 20-31% nullo-X, 7-16% nullo-4; plus homozygous *Scant* the values are 15-17% and 10-11%. So two doses of *Scant* does seem to be sufficient to reduce meiotic *polo^+^* enough to reduce *mtrm*-induced nondisjunction, just not as efficiently as halving the level of *polo^+^* directly. Again, fertility is reduced; now the control is homozygous *Scant*. Adding *mtrm* or *FM7* reduces fertility by half; adding both reduces it to 15%. This seems to imply that *mtrm* and *Scant* interact in producing a viable embryo.

One last feature of these data is that the control females from the *mtrm^126^* stock, but not those from the *mtrm^13^* stock, have elevated frequencies of exceptional progeny. Since all the first-generation females tested (Tables S1 and S2; numbers in [ ] indicate how many of the females tested gave exceptions) show this effect but only some of the third-generation females do (Tables S3 and S4), this is likely caused by an X- or second-linked dominant extraneous female meiotic mutation homozygous in this copy of the *mtrm^126^* stock; this is being followed up. However, comparing *mtrm^13^* and *mtrm^126^* in Tables S1-S4 suggests that *mtrm* is epistatic to it.
