# Supplementary material for: Mutations in Drosophila Greatwall/Scant Reveal Its Roles in Mitosis and Meiosis and Interdependence with Polo Kinase
Source: PLoS Genet. 2007 Nov 9;3(11):e200. doi: 10.1371/journal.pgen.0030200 (PMC2065886; doi:10.1371/journal.pgen.0030200)
Supplement: Supplementary file 4 [file pgen.0030200.sd003.doc]

### FlyBase Accession Numbers

*greatwall* (*gwl*; CG7719): FBgn0004461, *Scant*: FBal0056190

*polo* (CG12306): FBgn0003124, *polo^1^*: FBal0013924

*chk2/loki* (*lok;* CG10895): FBgn0019686, *lok^p6^*: FBal0143332

*matrimony* (*mtrm;* CG18543): FBgn0010431, *mtrm^126^*: FBal0175579, *mtrm^13^*: FBal0151716

### Supporting References

1. White-Cooper H, Carmena M, Gonzalez C, Glover DM (1996) Mutations in new cell cycle genes that fail to complement a multiply mutant third chromosome of Drosophila. Genetics 144: 1097-1111.

2. Xiang Y, Takeo S, Florens L, Hughes SL, Huo L-J, Gilliland WD, Swanson SK, Teeter K, Schwartz JW, Washburn MP, Jaspersen SL, Hawley RS (2007) The inhibition of polo kinase by matrimony facilitates G2 arrest in the meiotic cell cycle. PLoS Biol. In press.

3. Xu J, Du W (2003) Drosophila chk2 plays an important role in a mitotic checkpoint in syncytial embryos. FEBS Lett 545: 209-212.
