# Supplementary material for: Mutations in Drosophila Greatwall/Scant Reveal Its Roles in Mitosis and Meiosis and Interdependence with Polo Kinase
Source: PLoS Genet. 2007 Nov 9;3(11):e200. doi: 10.1371/journal.pgen.0030200 (PMC2065886; doi:10.1371/journal.pgen.0030200)
Supplement: Supplementary file 5 [file pgen.0030200.st001.doc]

**Table S1. Progeny from females with normal *X* chromosomes and the indicated third-chromosome genotype crossed to *YSX.YL, v f B/0; C(4)RM, ci eyR/0* males.** Parental crosses were *FM7/+; sr Scant* females x *y w/Y; mtrm^13^/TM313* or *y w/y^+^Y; mtrm^126^/TM3126* males and *FM7/Df(1)p116D, w* females by both genotypes of *mtrm* males. *y w/w Df* females from the latter cross produce only half the expected number of regular males, since the deficiency is recessive lethal; for these progeny, the number of regular males has been doubled (parentheses) before calculating gametic frequencies. Although diplo-*X* ova produce phenotypically distinct progeny here, they have been included in the calculations as though they could not be distinguished from mono-*X* ova so the frequencies here will be comparable to those in Table S4. Tests set up on different days are presented separately.

Third	No. Par.	B/+ Females	+ Males	(B^+^ Females)	v f B Males	Prog./	Gametic

Genotype	Females	+	ci ey	+	ci ey	+	ci ey	+	ci ey	Female	nullo-X	nullo-4

*sr Scant/TM313*	3	19	0	61	0	0	0	0	0	26.7	0	0

*sr Scant/TM3126*	4[4]	55	10	198	39	(1)	0	0	0	75.8	0	0.162

*y w/w Df; mtrm^13^/+*	9	467	52	382	30	(28)	(13)	36	3	112(158)	0.053	0.090

10	525	51	334	33	(33)	(14)	57	1	105(142)	0.079	0.090

*y w/w Df; mtrm^126^/+*	9	485	34	333	16	(48)	(10)	44	1	108(147)	0.066	0.057

4	166	30	91	11	(16)	(2)	26	7	87(113)	0.136	0.140

*mtrm^13^/Scant*	7	220	22	405	31	(14)	(8)	12	1	101.9	0.036	0.087

10	314	22	537	32	(17)	(7)	20	7	95.6	0.055	0.076

*mtrm^126^/Scant*	8	174	24	328	41	(28)	(6)	17	8	78.2	0.077	0.134

9	255	43	441	62	(35)	(1)	22	9	96.4	0.069	0.138
