# Supplementary material for: Mutations in Drosophila Greatwall/Scant Reveal Its Roles in Mitosis and Meiosis and Interdependence with Polo Kinase
Source: PLoS Genet. 2007 Nov 9;3(11):e200. doi: 10.1371/journal.pgen.0030200 (PMC2065886; doi:10.1371/journal.pgen.0030200)
Supplement: Supplementary file 6 [file pgen.0030200.st002.doc]

**Table S2.** **Progeny from *FM7/+* females with the indicated third-chromosome genotype crossed to *YSX.YL, v f B/0; C(4)RM, ci eyR/0* males.** Females tested are sibs of those in Table S1. *FM7/0* males are nearly always lethal.

Third	No. Par.	B/+, B/B Fem.	+ Males	(y2 wa B/+ Fem.)	v f B Males	Prog./	Gametic

Genotype	Females	+	ci ey	+	ci ey	+	ci ey	+	ci ey	Female	nullo-X	nullo-4

*sr Scant/TM313*	4	7	0	7	0	(1)	0	0	0	3.8	0	0

*sr Scant/TM3126*	5[≥3]	6	1	18	1	(2)	(1)	2	0	6.2	0.121	0.091

*mtrm^13^/+*	10	337	67	363	48	(104)	(40)	171	8	113.8	0.271	0.130

4	93	21	89	18	(41)	(9)	53	7	82.8	0.307	0.159

*mrm^126^/+*	7	208	18	184	13	(75)	(15)	95	2	87.1	0.274	0.070

7	186	15	187	16	(80)	(14)	57	6	80.1	0.202	0.091

*mtrm^13^/Scant*	5	56	2	54	4	(33)	(5)	32	6	38.4	0.330	0.100

5	46	6	64	6	(35)	(11)	28	8	40.8	0.300	0.162

*mtrm^126^/Scant*	3	37	4	47	0	(17)	(5)	12	1	41.0	0.191	0.081

4	65	3	50	4	(47)	(4)	28	6	51.8	0.282	0.095
