# Supplementary material for: Mutations in Drosophila Greatwall/Scant Reveal Its Roles in Mitosis and Meiosis and Interdependence with Polo Kinase
Source: PLoS Genet. 2007 Nov 9;3(11):e200. doi: 10.1371/journal.pgen.0030200 (PMC2065886; doi:10.1371/journal.pgen.0030200)
Supplement: Supplementary file 7 [file pgen.0030200.st003.doc]

**Table S3. Progeny from females with normal *X* chromosomes and the indicated third-chromosome genotype crossed to *YSX.YL, v f B/0; C(4)RM, ci eyR/0* males.** Parental crosses were *FM7/+; sr Scant* females *x Scantf/TM6Bf, mtrm^13^ Scant/TM6B13,* or *mtrm^126^ Scant/TM6B126* males.

Third	No. Par.	B/+ Females	+ Males	(B^+^ Females)	v f B Males	Prog./	Gametic

Genotype	Females	+	ci ey	+	ci ey	+	ci ey	+	ci ey	Female	nullo-X	nullo-4

*sr Scant/TM6Bf*	5	150	0	163	0	0	0	0	0	62.6	0	0

*sr Scant/TM6B13*	6	180	0	152	0	0	0	0	0	55.3	0	0

*sr Scant/TM6B126*	5[3]	163	6	164	4	0	0	0	0	67.4	0	0.030

*Scantf/sr Scant*	16	193	0	427	0	0	0	0	0	38.8	0	0

*mtrm^13^ Scant/sr Scant*	57	178	7	575	14	(24)	(3)	12	3	14.3	0.036	0.036

*mtrm^126^ Scant/sr Scant*	66	330	14	829	34	(37)	(4)	26	7	19.4	0.050	0.050
