# Supplementary material for: Mutations in Drosophila Greatwall/Scant Reveal Its Roles in Mitosis and Meiosis and Interdependence with Polo Kinase
Source: PLoS Genet. 2007 Nov 9;3(11):e200. doi: 10.1371/journal.pgen.0030200 (PMC2065886; doi:10.1371/journal.pgen.0030200)
Supplement: Supplementary file 8 [file pgen.0030200.st004.doc]

**Table S4. Progeny from *FM7/+* females with the indicated third-chromosome genotype crossed to *YSX.YL, v f B/0; C(4)RM, ci eyR/0* males**. Females tested are sibs of those in Table S3.

Third	No. Par.	B/+ Females	v B/B females	+ Males	v f B Males	Prog./	Gametic

Genotype	Females	+	ci ey	+	ci ey	+	ci ey	+	ci ey	Female	nullo-X	nullo-4

*sr Scant/TM6Bf*	5	66	0	34	0	58	0	8	0	33.2	0.092	0

*sr Scant/TM6B13*	9	98	1	72	0	108	1	9	0	32.1	0.060	0.007

*sr Scant/TM6B126*	6[1]	69	1	47	3	103	2	14	0	39.8	0.111	0.024

*Scantf/sr Scant*	14	35	0	35	0	155	0	0	0	16.1	0	0

*mtrm^13^ Scant/sr Scant*	47	64	8	26	1	101	5	16	5	4.81	0.170	0.097

*mtrm^126^ Scant/sr Scant*	50	142	11	42	3	113	7	17	11	6.92	0.150	0.115
